# Can incorporating genotyping data into efficacy estimators improve efficiency of early phase malaria vaccine trials?

**DOI:** 10.1186/s12936-023-04802-0

**Published:** 2023-12-19

**Authors:** Gail E. Potter, Viviane Callier, Biraj Shrestha, Sudhaunshu Joshi, Ankit Dwivedi, Joana C. Silva, Matthew B. Laurens, Dean A. Follmann, Gregory A. Deye

**Affiliations:** 1 Biostatistics Research Branch, National Institute of Allergy and Infectious Diseases, National Institutes of Health, Rockville, MD USA; 2 Clinical Monitoring Research Program Directorate, Frederick National Laboratory for Cancer Research, Frederick, MD USA; 3 Center for Vaccine Development and Global Health, University of Maryland School of Medicine, Baltimore, MD USA; 4 Institute for Genomic Sciences, University of Maryland School of Medicine, Baltimore, MD USA; 5 Department of Microbiology & Immunology, University of Maryland School of Medicine, Baltimore, MD USA; 6 Division of Microbiology and Infectious Diseases, National Institute of Allergy and Infectious Diseases, National Institutes of Health, Rockville, MD USA; 7 Present Address: AstraZeneca PLC, Gaithersburg, MD USA

**Keywords:** Vaccine efficacy, Efficacy, Genotyping, Clone, Molecular force of infection, Molecular endpoints, Early phase, Trial efficiency, Efficiency

## Abstract

**Background:**

Early phase malaria vaccine field trials typically measure malaria infection by PCR or thick blood smear microscopy performed on serially sampled blood. Vaccine efficacy (VE) is the proportion reduction in an endpoint due to vaccination and is often calculated as VE_HR_ = 1–hazard ratio or VE_RR_ = 1–risk ratio. Genotyping information can distinguish different clones and distinguish multiple infections over time, potentially increasing statistical power. This paper investigates two alternative VE endpoints incorporating genotyping information: VE_molFOI_, the vaccine-induced proportion reduction in incidence of new clones acquired over time, and VE_C_, the vaccine-induced proportion reduction in mean number of infecting clones per exposure.

**Methods:**

Power of VE_molFOI_ and VE_C_ was compared to that of VE_HR_ and VE_RR_ by simulations and analytic derivations, and the four VE methods were applied to three data sets: a Phase 3 trial of RTS,S malaria vaccine in 6912 African infants, a Phase 2 trial of PfSPZ Vaccine in 80 Burkina Faso adults, and a trial comparing *Plasmodium vivax* incidence in 466 Papua New Guinean children after receiving chloroquine + artemether lumefantrine with or without primaquine (as these VE methods can also quantify effects of other prevention measures). By destroying hibernating liver-stage *P. vivax*, primaquine reduces subsequent reactivations after treatment completion.

**Results:**

In the trial of RTS,S vaccine, a significantly reduced number of clones at first infection was observed, but this was not the case in trials of PfSPZ Vaccine or primaquine, although the PfSPZ trial lacked power to show a reduction. Resampling smaller data sets from the large RTS,S trial to simulate phase 2 trials showed modest power gains from VE_C_ compared to VE_HR_ for data like those from RTS,S, but VE_C_ is less powerful than VE_HR_ for trials in which the number of clones at first infection is not reduced. VE_molFOI_ was most powerful in model-based simulations, but only the primaquine trial collected enough serial samples to precisely estimate VE_molFOI_. The primaquine VE_molFOI_ estimate decreased after most control arm liver-stage infections reactivated (which mathematically resembles a waning vaccine), preventing VE_molFOI_ from improving power.

**Conclusions:**

The power gain from the genotyping methods depends on the context. Because input parameters for early phase power calculations are often uncertain, these estimators are not recommended as primary endpoints for small trials unless supported by targeted data analysis.

*Trial registrations:* NCT00866619, NCT02663700, NCT02143934.

**Supplementary Information:**

The online version contains supplementary material available at 10.1186/s12936-023-04802-0.

## Background

Phase 3 malaria vaccine trials generally use clinical malaria as an endpoint as this is a relevant measure of how a patient “feels, functions, or survives” For small, early phase trials in malaria-experienced populations, rates of clinical malaria may be too low to have sufficient power. Outcomes with higher event rates, such as malaria infection detected by thick blood smear microscopy or PCR testing on serial blood samples are more feasible. Vaccine efficacy is often measured as VE_HR_ = 1–hazard ratio [2] or VE_RR_ = 1–risk ratio. The HR approach is generally more powerful than the risk ratio approach since it incorporates information on the timing of events and allows detection of a treatment effect even when all participants become infected during follow-up. It is more informative for a vaccine that is “leaky” (conferring partial protection on all individuals) than “all-or-nothing” (conferring complete protection on some individuals and no protection on others) [3, 4].

An alternative approach analyses multiple infections per person during follow-up by comparing the incidence rates of infections between vaccine and control arms. Counting distinct infections is challenging when malaria infection is measured by thick blood smear microscopy performed on serially sampled blood because this information is insufficient to determine whether two consecutive positive results are distinct infections. Genotyping can be used to distinguish different infections over time and can also count synchronous infection with different strains as multiple infections. The number of new clones acquired per year has been referred to as the “molecular force of infection” (molFOI) and was correlated with clinical *Plasmodium falciparum* malaria (defined as febrile illness plus *Pf* parasitaemia > 2500/μL) in Papua New Guinean children [5]. Thus, an alternative efficacy measure is the proportion reduction in molFOI due to vaccination, measured as

$$\textrm{VE}_{\textrm{molFOI}} \, = \,1\, - \,\frac{\text{molFOI of vaccinees}}{\text{{molFOI of controls}}}$$

This efficacy measure has been proposed as a new measure for vaccine efficacy trials [6, 7], but it has not been applied or tested in clinical vaccine trials, although it has been applied in observational studies: one study compared molFOI and infection clearing times between males and females to better understand higher malaria prevalence in males in cross-sectional samples [8], and another used molFOI in exploring possible mechanisms by which sickle cell trait (HbAS) protects against *P. falciparum* malaria [9].

VE_molFOI_ has the potential to increase power by incorporating information from multiple events, but it discards information from the timing of infections. A second efficacy measure incorporating genotyping information incorporates both time-to-event information and the number of clones present at the first infection. This measure was developed in the context of HIV (with virions measured instead of clones) [10] and has been applied to a Phase 3 trial of RTS,S malaria vaccine in 6912 infants [11, 12], which was also analysed in this paper. Although the authors denoted this measure VE_V_ (V for “virion”), in this paper it is denoted VE_C_ (C for “clone”). VE_C_ is defined as the proportion reduction in number of infecting clones per exposure, due to vaccination. “Exposure” is the instantaneous exposure over time and is modelled as a completely flexible (unspecified) function of time. The only assumption is that the exposure function is identical in vaccine and control arms, which is reasonable for randomized controlled trials. To define VE_C_, let X* denote the number of inoculated clones from a given exposure that develop into a blood stage infection, and let Z be a treatment indicator variable, so Z = 1 for vaccinees and Z = 0 for controls. Then, letting $\Delta =\frac{E\left({X}^{*}|Z=1, exposed\right)}{E\left({X}^{*}|Z=0, exposed\right)}$, the efficacy measure is:

$${VE}_{C}=1-\Delta = 1- \frac{E\left({X}^{*}|Z=1, exposed\right)}{E\left({X}^{*}|Z=0, exposed\right)}$$

Zero values of X* are not observed because exposure events are not observed in malaria field trials, which only detect clones that develop into blood stage infections. Let X denote the observed number of clones, so X is a truncated version of X* such that X > 0. Through algebra, Follmann and Huang showed that VE_C_ can be estimated as:

$${\widehat{VE}}_{C}= 1-\frac{{\overline{x} }_{v}}{{\overline{x} }_{c}}\mathrm{HR},$$

where ${\overline{x} }_{v}$ and ${\overline{x} }_{c}$ are the mean number of clones at first infection in vaccinees and controls, respectively. While the estimand $\Delta$ is a ratio of untruncated population means, its estimator is a ratio of truncated means times the HR for the time to the first exposure that causes an infection. The intuition behind this is that if fewer exposures lead to infection in the vaccine group, then there are more unobserved zeroes in that group, which is mathematically reflected in the HR < 1 for the first infection. Note that for this measure, the numbers of clones are measured at the time of first infection. However, in contrast to a simple comparison of mean number of clones at first infection, VE_C_ analyses all participants (both infected and uninfected). VE_C_ takes into account both the timing of the first infection and the number of clones for that infection.

**Fig. 1:**
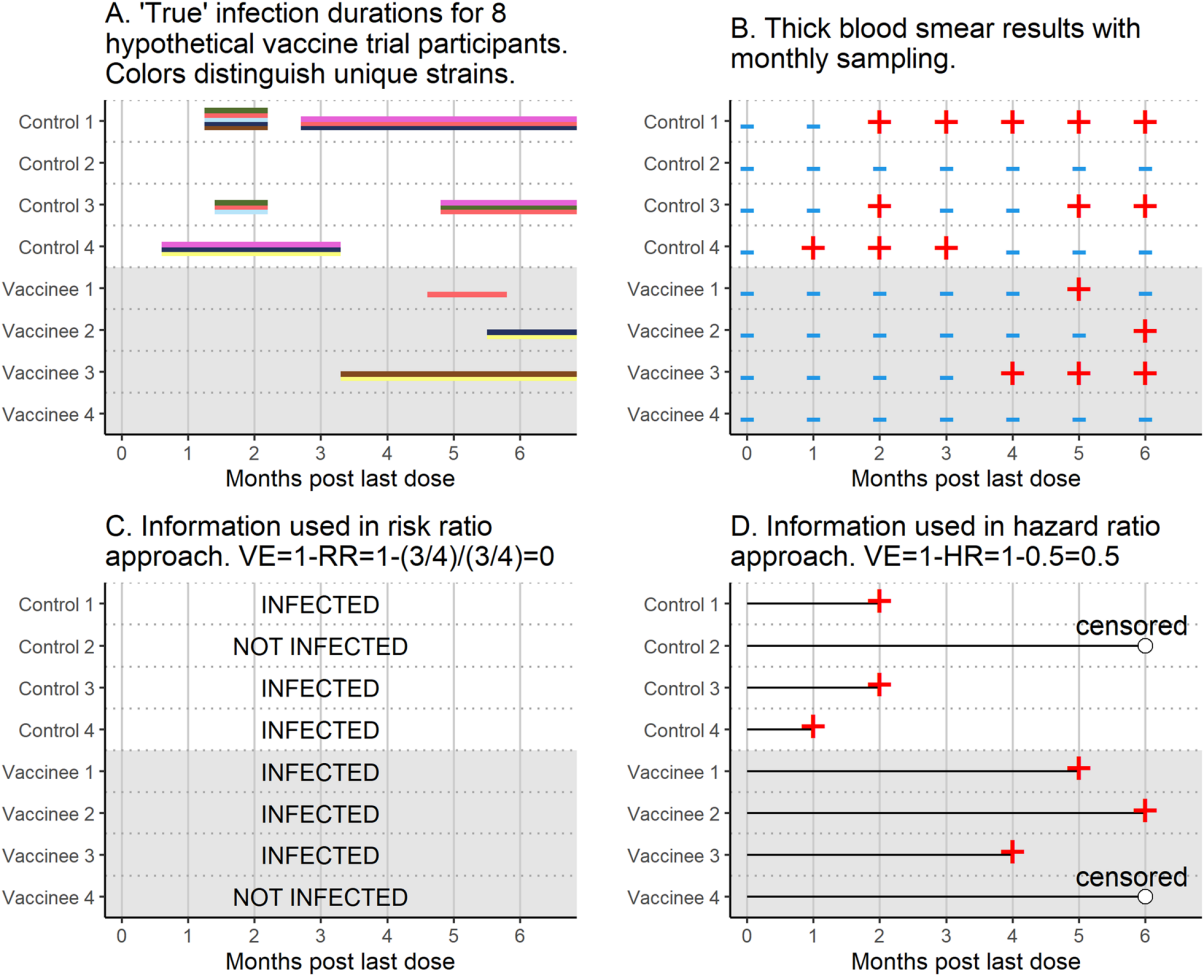
Infection durations for 8 hypothetical trial participants (Panel** A**), information recorded by monthly sampling (Panel** B**), information incorporated into VE_RR_ (Panel** C**), and information incorporated into VE_HR_ (Panel** D**)

**Fig. 2:**
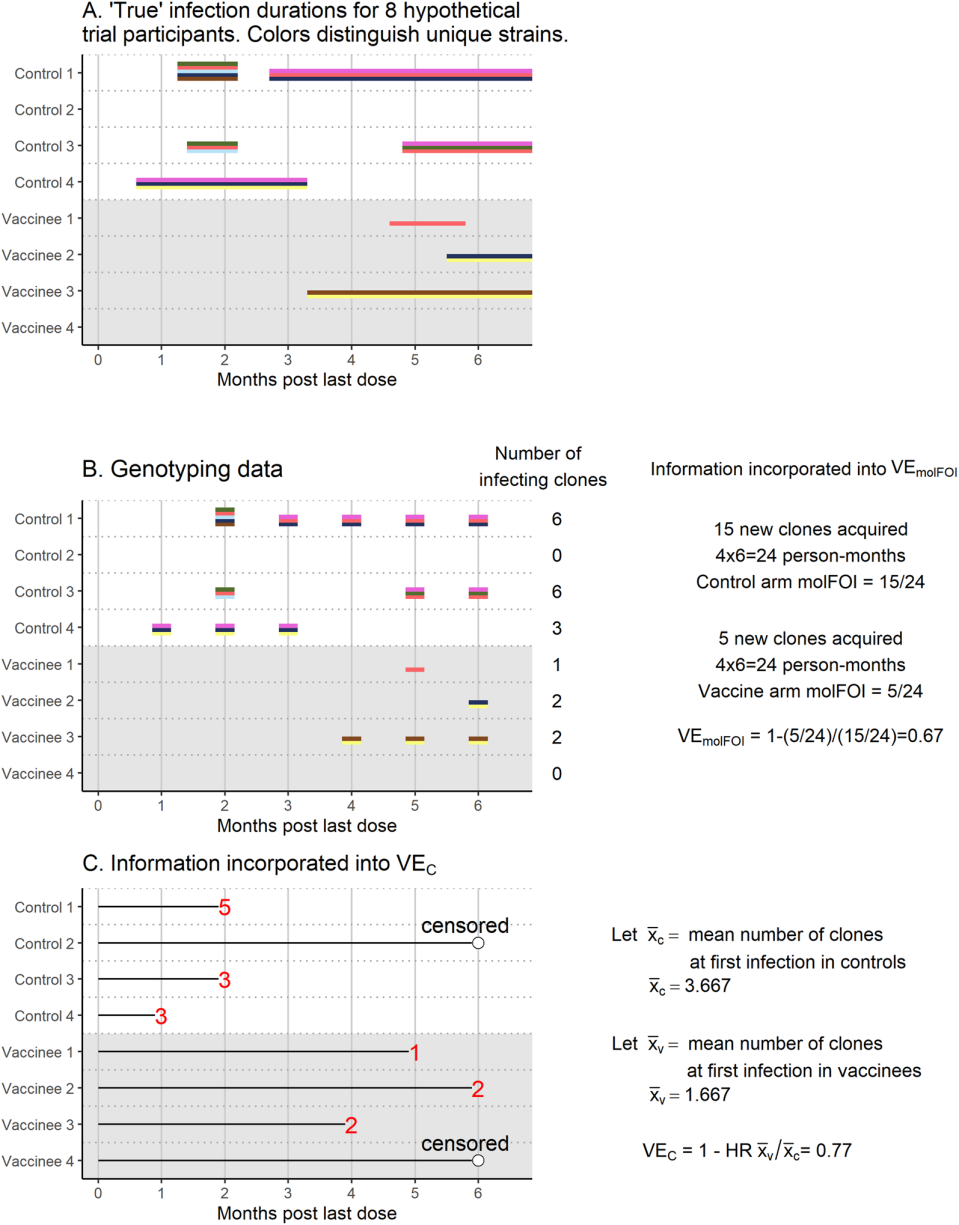
Infection durations for 8 hypothetical trial participants (Panel** A**), observed genotyping data and information contributing to VE_molFOI_ (Panel** B**), and information incorporated into VE_C_ (Panel** C**)

Figures 1 and 2 illustrate information used in the four VE methods for a toy example. Figure 1 shows information used in the two standard methods, VE_RR_ and VE_HR_. Panel A shows “true” infection durations for 8 hypothetical vaccine trial participants, with colours distinguishing different clones, and Panel B shows information gathered when thick blood smear microscopy is performed on monthly samples. VE_RR_ uses the presence/absence of infection during follow-up, while VE_HR_ uses the time to first infection. Figure 2 shows the calculation of the two VE measures incorporating genotyping data, VE_molFOI_ and VE_C_. Panel A is included again for reference, and Panel B shows calculation of VE_molFOI_. In this example, repeat observations of the same clone are counted as new infections if they were separated by at least one sample for which that clone was not observed. Panel C shows calculation of VE_C_, which uses the time to first infection and number of clones present at first infection.

This paper systematically explores the feasibility of these two VE measures in small trials by testing their operating characteristics in a comprehensive set of simulation studies and applying them to data from three randomized, placebo-controlled trials. The mean, variability, and statistical power of the VE measures are compared to standard VE estimators in model-based simulations and by resampling. Implications of their adoption for the trial pipeline are discussed. Although this paper refers to these measures as VE (for “vaccine efficacy”), they can equally be considered and applied to trials of other malaria prevention measures.

## Data

The following three data sets were analysed:

1. ***RTS,S data:*** In this Phase 3 trial, participants in 7 African countries enrolled from March 2009 – Jan 2011 and were randomized 2:1 to receive either 3 doses of RTS,S/AS01 malaria vaccine or placebo [11] (NCT00866619). Clinical malaria from *P. falciparum* infection was tracked for 12 months. Thick blood smear microscopy was performed on samples collected at the first clinical malaria episode, defined as temperature of 37.5 °C or higher and *P. falciparum* asexual parasitaemia at more than 5000 parasites/μL or severe malaria. Serial blood samples were not collected. The data set analyzed in this paper comprised 6912 participants aged 5–17 months who completed their dosing regimen and were included in the per-protocol population [12, 13]. Of these, 2391 developed clinical malaria during the follow-up period, and genotyping results were obtained for 2089 (87%) of these first clinical malaria episodes. 908 (39%) of controls and 1181 (26%) of vaccinees experienced clinical malaria with non-missing genotyping results.
2. ***PfSPZ data:*** This Phase 2 trial included 80 Burkina Faso adults who enrolled in March 2017 and were randomized 1:1 to receive 3 doses of PfSPZ Vaccine or placebo [14] (NCT02663700). Thick blood smear microscopy was performed on blood samples collected monthly and when ill with malaria symptoms to detect *P. falciparum* infection, and genotyping was performed on the first positive sample only. There were 37 first infections by thick blood smear microscopy: 14 of 39 (36%) vaccinees and 23 of 41 (56%) controls. Genotyping results were obtained for 33 of the 37 first infections (89%).
3. ***Primaquine data:*** This randomized controlled trial tracked molecular force of *Plasmodium vivax* blood stage infection in children randomized 1:1 to receive either blood + liver stage treatment (chloroquine (CQ) + artemether-lumefantrine (AL) + primaquine (PQ)) or blood stage treatment only (CQ + AL + placebo) at enrollment [15] (NCT02143934). *P. vivax* parasites can hibernate unobserved in the human liver (without causing symptoms in their host) for months or years and then “reactivate” to cause an observable and potentially symptomatic blood stage infection. The goal of primaquine treatment was to clear hibernating *P. vivax* parasites from the liver*.* Thus, *P. vivax* infections in controls included relapses and new infections, while in treated participants, new infections were assumed to be unchanged while a portion of relapses were prevented. The aim of the trial was to test whether the addition of primaquine to the CQ + AL regimen at baseline could reduce *P. vivax* infections in the subsequent 8-month follow-up period. 529 children aged 5–10 years enrolled from 17 August to 11 September 2009 in six villages in Maprik district, East Sepik Province, Papua New Guinea. Treatment was given over 28 days, with CQ for the first three days, PQ for 5 days per week, and AL for Days 11–13. After the end of the treatment period, blood was collected biweekly for 3 months, then monthly for 5 more months. The data set analysed in this paper includes genotyping data from the 466 children who completed the full course of treatment [16, 17]. Of these, 48% tested positive for *P. vivax* by PCR before study treatment, and 1% tested positive after treatment. The extent of missing genotyping results is unclear because the public data set provides limited information: it reports *P. vivax* parasitaemia by PCR and the number of new *P. vivax* clones detected during an interval, so a positive *P. vivax* PCR can be consistent with zero new *P. vivax* clones (if the clones detected were previously observed). However, 31 (14%) of 229 first post-baseline infections were PCR-positive for *P. vivax* and had zero new *P. vivax* clones detected—an inconsistency between the two tests. Thus, one might expect that for about 14% of subsequent *P. vivax* PCR positive results, genotyping failed to detect the *P. vivax* clone that was present.

## Methods

Table 1 summarizes the four VE methods studied in this paper. The first two are standard approaches (Fig. 1), while the second two incorporate molecular data (Fig. 2). These four methods are tested in simulation and in data analysis.

**Table 1:** VE (vaccine efficacy) methods, interpretation, and formulas

Method	Meaning	Formula
VE_RR_	Vaccine-induced proportion reduction in probability of infection during follow-up	${\mathrm{VE}}_{\mathrm{RR}}= 1-\mathrm{RR}= 1-\frac{{\mathrm{p}}_{\mathrm{v}}}{{\mathrm{p}}_{\mathrm{c}}}$
VE_HR_	Vaccine-induced proportion reduction in hazard of first infection during follow-up	${\mathrm{VE}}_{\mathrm{HR}}=$ 1 – HR
VE_molFOI_	Vaccine-induced proportion reduction in molecular force of infection (molFOI)	VE_molFOI_ = $1- \frac{\mathrm{molFOI\, of\, vaccinees}}{\mathrm{molFOI\, of\, controls}}$
VE_C_	Vaccine-induced proportion reduction in mean number of clones per exposure that develop into a blood stage infection	${\mathrm{VE}}_{C}= 1-\frac{{\overline{x} }_{v}}{{\overline{x} }_{c}}\mathrm{HR}$

RR,  risk ratio; ${p}_{v}$, proportion of vaccinees infected; ${p}_{c}$, proportion of controls infected;

HR,  hazard ratio for time to first infection

molFOI, incidence of new clones acquired over time = $$\frac{\begin{array}{c}Number \,of \,new \,clones \,acquired over \,follow-up, \,summed\\ over \,all \,members \,of \,the \,group\end{array}}{\mathrm{Total \,time \,in \,follow}-\mathrm{up \,in \,group}}$$

${\overline{x} }_{v}$, number of clones at first infection in vaccines; ${\overline{x} }_{c}$, number of clones at first infection in controls

## Simulation study 1

To compare power between the four VE methods, a malaria vaccine randomized controlled trial was simulated with 1:1 randomization and a 168-day follow-up period. Exposure events with equal rates in the two arms were simulated via a Poisson process, which is equivalent to sampling exponential times to exposure for each person and allowing multiple events per person during follow-up. An “exposure event” was defined as exposure sufficient to cause a blood-stage infection in a control participant, so the event may correspond to multiple infectious bites. Note that this is different from the standard conceptualization of exposure, which does not always lead to an infection in unvaccinated people. Each exposure event may transfer multiple *P. falciparum* clones, an assumption based on work suggesting that co-transmission of multiple clones from a single mosquito is more common than superinfection (infection with multiple clones from different mosquitoes) [18, 19]. Each clone may then be blocked by the vaccine. The simulations assumed that 10 clones are circulating in the community, and for each exposure event, the number of clones transferred (n_c_) was sampled from a Poisson random variable truncated to lie between 1 and 10. Separate simulations were performed for a mean of 1, 2, or 3 clones transferred per exposure. Then, the identities of the n_c_ clones transferred during the bite were sampled from the set of 10 circulating clones with equal probability. Different simulations implemented different vaccine blocking mechanisms:

1. Scenario 1: Each clone was blocked independently with probability 0.5.
2. Scenario 2: A prespecified set including half of the circulating clones were blocked with probability 100% (when transferred to vaccinees), and the other half were never blocked.
3. Scenario 3: The prespecified set of half of the circulating clones were blocked with probability 75% (when transferred to vaccinees), and the other half were blocked with probability 25%.
4. Scenario 4: To estimate Type 1 error (the probably of incorrectly rejecting the null hypothesis when the null hypothesis is true), an ineffective vaccine was simulated.

For each clone that developed into a blood stage infection, an infection duration was sampled from an exponential distribution with a mean of 303 days for females and 167 days for males [8]. Biweekly sampling for 168 days was simulated, and hypothesis tests for the four VE measures for each simulated trial were performed. Power was estimated as the proportion of simulations in which the null hypothesis was rejected. Further details are in the Additional file (Additional file 1: Supplementary Methods Sect. 1). The simulation was repeated with 500 circulating clones instead of 10 circulating clones, but still assumed that a maximum of 10 clones could be transferred per exposure.

### Data analysis

For each of the four VE methods, an efficacy estimate, 95% confidence interval (CI), and p-value were calculated for each data set. Risk ratios were estimated by modified Poisson regression [20] in the primaquine and RTS,S vaccine trials. This approach models the outcome as binary (infected vs. not infected) using a Poisson distribution, which gives a valid estimate of the risk ratio. Since a Poisson distribution is not a good fit for the Bernoulli distribution, variance estimates would be inaccurate by this approach, a problem corrected through the use of robust “sandwich” standard errors [20]. In the PfSPZ Vaccine trial, the risk ratio was estimated based on Kaplan Meier estimated survival probabilities, as this method was prespecified as the primary analysis method. Their variances were calculated by Greenwood’s formula, and the delta method was applied to obtain the VE CI [21]. In all trials, hazard ratios were from Cox regression with Wald tests, and ${\mathrm{VE}}_{\mathrm{C}}$ was calculated by estimating $\Delta$ as $\frac{{\overline{x} }_{v}}{{\overline{x} }_{c}}\mathrm{HR}$, so ${\widehat{\mathrm{VE}}}_{\mathrm{C}}= 1-\frac{{\overline{x} }_{v}}{{\overline{x} }_{c}}\mathrm{HR}.$ Analogous to Cox regression, $\Delta$ is estimated on the log scale. The standard error for the estimator for log($\Delta$) is given by the below formula [10]:

$${\mathrm{SE}}_{\mathrm{log}\widehat{\Delta }}= \sqrt{{{\mathrm{SE}}_{\widehat{\mathrm{logHR}}}}^{2}+\frac{{\mathrm{s}}_{\mathrm{v}}^{2}}{{\mathrm{I}}_{\mathrm{v}}{{\overline{\mathrm{x}} }_{\mathrm{v}}}^{2} }+\frac{{\mathrm{s}}_{\mathrm{c}}^{2}}{{\mathrm{I}}_{\mathrm{c}}{{\overline{\mathrm{x}} }_{\mathrm{c}}}^{2} },}$$

where ${\mathrm{SE}}_{\widehat{\mathrm{logHR}}}$ is the standard error of the log hazard ratio from Cox regression, ${\mathrm{s}}_{\mathrm{v}}^{2}$ and ${\mathrm{s}}_{\mathrm{c}}^{2}$ denote the variances of number of clones at first infection in vaccinees and controls, respectively, and I_v_ and I_c_ denote numbers of vaccinees and controls infected. Confidence intervals and p-values for ${\mathrm{VE}}_{\mathrm{C}}$ are calculated based on normality of the test statistic. In all trials, the ratio of molecular force of infection was estimated by quasi-Poisson regression with the total number of clones acquired over follow-up per person as the response variable and the log-transformed time-at-risk as an offset.

Time-at-risk was the end of follow-up in the primaquine data set because the genotyping results from serially sampled blood allow observation of repeat infections. Intervals of 42 days or more with no study visits were subtracted from time-at-risk for consistency with previous analyses [16]. In the other two data sets, time-at-risk was truncated at the first infection since genotyping results from subsequent infections were not obtained. In calculating time-at-risk, time receiving antimalarial medication was not subtracted. Because time-at-risk in the RTS,S and PfSPZ Vaccine trials was based on the first malaria infection, there was likely little or no anti-malarial medication received during the at-risk interval. In the primaquine trial, this analysis approach was taken for consistency with prior analyses. Although medication data during the follow-up period is not available in the public data set, clinical episodes (defined as fever and a positive malaria infection by light microscopy) are in the data set, and there were few in this trial: 27 controls (12%) and 18 PQ recipients (8%) experienced clinical malaria. In the primaquine data set, 31 (14%) first infections were PCR positive for *P. vivax* but had no *P. vivax* clones reported. These were analysed as zero clones when calculating VE_molFOI_. Of these 31, 27 had zero and 4 had missing new clones; these were analysed as zero and missing, respectively, when calculating the mean number of clones at first infection. A sensitivity analysis was performed imputing each of these events to have 1 new *P. vivax* clone. In the Burkina Faso data set, 4 first infections were missing a genotyping result. These were analysed as no new clones when calculating VE_molFOI_ and as missing when calculating the mean number of clones at first infection. In the RTS,S trial, only clinical malaria events with non-missing genotyping information were analysed, for consistency with previous analyses [12].

## Simulation study 2

The purpose of this simulation is to assess whether the three data sets are consistent with the model underlying the simulation process. The model operationalizes the vaccine mechanism as a per-clone blocking probability. From this, reductions in all outcomes (risk of infection, hazard ratio, mean number of clones per infection, and molFOI) are affected simultaneously, so treatment effects on all outcomes are correlated. In this second simulation study, the simulation inputs were recalibrated to match the probability of at least one infection during follow-up in each group and the mean number of clones per infection in the control group for each of the three trials analysed. The inputs adjusted were the exposure rate, the mean number of clones transferred per exposure, and the clone blocking probability. The mean number of clones per infection in the vaccine group is induced by the distribution of clones in the control group, the clone blocking probability, and the blocking mechanism. The exposure rate was mathematically derived (assuming exponential time-to-event distributions) so that the simulated control group infection rate matched the observed rate. Blocking probabilities were then estimated by looping through different blocking probability values until the simulated treatment group event rate matched the observed rate. Table 2 shows these infection rates for each trial and the values of input parameters used to simulate them. The actual total number of clones circulating in the community was not available, so 50 circulating clones were assumed with a maximum of 10 clones transferred per exposure. For each set of inputs, 1000 trials of size 500 were simulated, and the average VE estimator values, infection rates in each arm, and the mean and variance of the number of clones at first infection in each arm were tracked.

**Table 2:** Target infection rates and inputs used to generate them in Simulation Study 2

	Parameter	RTS,S/AS01 trial	Primaquine trial	PfSPZ Vaccine trial
Infection rates observed in each trial	Proportion of controls infected during follow-up	0.39	0.71	0.56
	Proportion of subjects in treatment arm infected during follow-up	0.26	0.28	0.36
Inputs to simulation study	Exposure rate	1/741	1/182	1/204
	Clone blocking probability	0.61	0.80	0.76
	Mean number of clones at first infection in control group	2.26	1.46	3.00
	Follow-up duration	12 months (364 days)	8 months (224 days)	6 months (168 days)

## Simulation study 3 and analytic power

Because the Phase 3 RTS,S data set is very large, it provides a population from which smaller trials can be resampled, which are hypothetical early phase trials. This third simulation study entailed resampling 10,000 trials of size 80, 150, and 250 from the RTS,S data set with equal numbers of participants in each arm. VE_HR_ and VE_C_ were calculated with 95% confidence intervals, and power was estimated as the proportion of simulations for which the interval excluded zero. Since the RTS,S trial had a lower event rate than what is usually seen in early phase trials, another resampling study was performed imposing a higher event rate by first simulating infection status for each control by tossing a weighted coin with infection probability 0.6, and doing the same for vaccinees with infection probability 0.3. For each infected vaccinee, the paired values of the time-to-event and number of clones at first event were sampled from the set of infected RTS,S vaccinees. For each uninfected vaccinee, the time to censoring was sampled from the set of uninfected RTS,S vaccinees. Values were sampled analogously for controls. To provide further context for Simulation Study 3, analytic power formulas were derived for two of the efficacy methods: VE_HR_ and VE_C_ and are in the Additional file 1: Supplementary Methods Sect. 2. These experiments and derivations were not performed for VE_molFOI_ because the information in the RTS,S data set for this estimator is limited due to the lack of serial sampling.

## Results

**Fig. 3:**
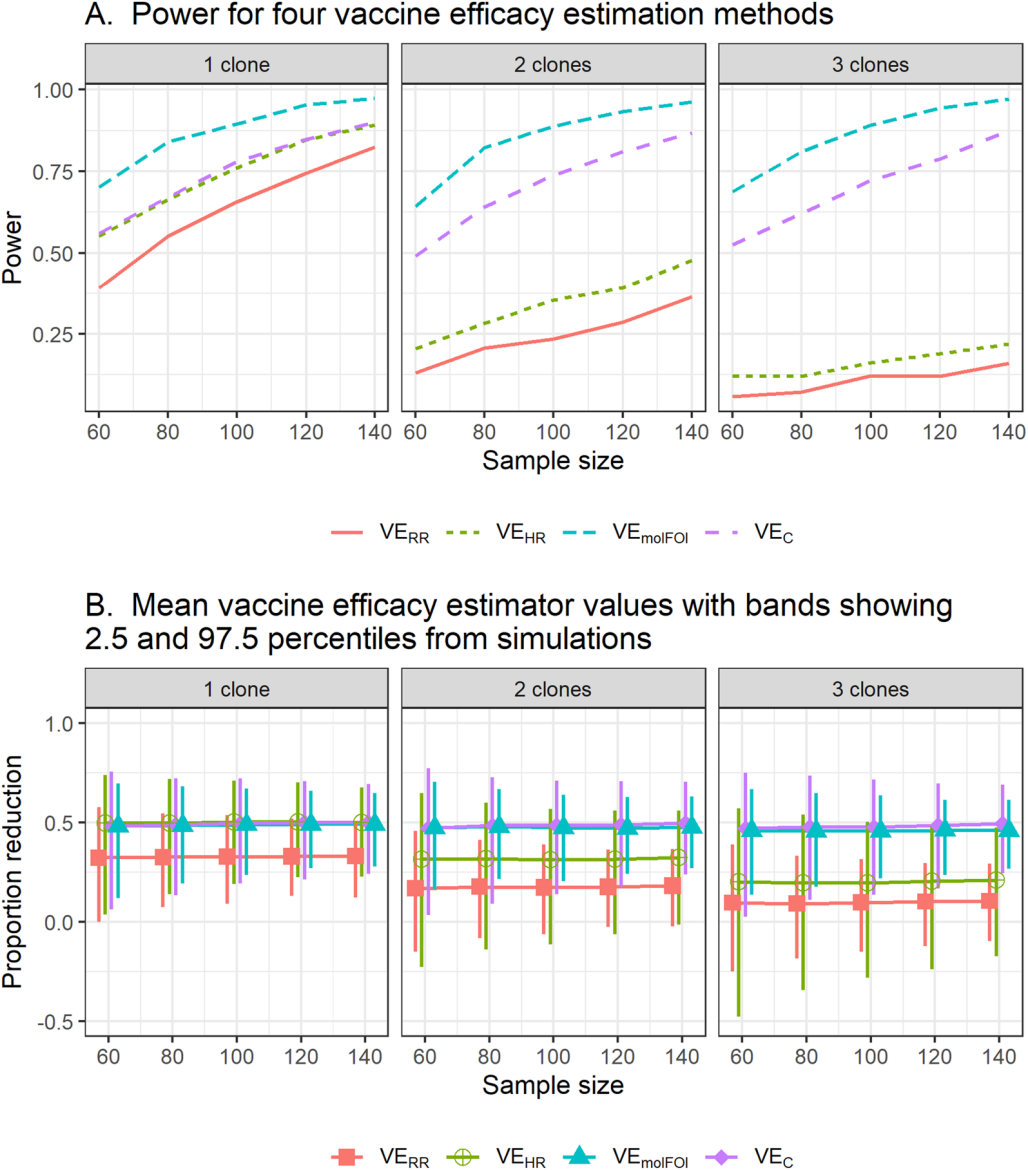
Estimated power by the four different VE methods by mean number of clones transferred per exposure (Panel** A**) and mean VE estimator values across simulations (Panel** B**) with variability represented by 2.5th and 97.5th percentiles. VE methods were calculated for the same sample sizes; the displayed intervals are staggered to distinguish them visually

Simulation Study 1 found that Type 1 error is controlled for all methods and shows similar power for each method under the three vaccine-blocking scenarios (Additional file 1: Fig. A1). This is because while the different scenarios implement different preferential blocking for different clones, the mean number of clones is reduced by the same amount. An exception is that for small sample sizes and large average number of clones transferred per exposure, the molecular force of infection method is more powerful in Scenario 2 (perfect blocking of half the clones and no blocking of the other half) than in other scenarios. That is because 10 circulating clones were assumed and reducing this to 5 among vaccinees reduces the variability of the number of clones, which reduces the variability of the VE estimator. Because the three scenarios of interest gave similar power, power is displayed only for Scenario 1 in Fig. 3 to facilitate comparison between methods. The simulations result in an infection rate of 75% in the control arm and 50% in vaccinees when a single clone is transferred per exposure. All panels show that the hazard ratio approach is more powerful than the risk ratio approach.

When a single clone is transferred per exposure, VE_HR_ and VE_C_ perform identically since the mean number of clones at first infection is 1 in both groups. When 2 clones are transferred per exposure (on average), the value of the HR is reduced because the vaccine must block two clones (each with probability 0.5) to prevent an infection, so VE_HR_ loses power, as does VE_RR_, but the genotyping methods do not lose power. This pattern continues when the mean number of clones per exposure is increased to 3. The lower panel shows that the expected values of estimators are constant across sample sizes, but variability of the estimator decreases as the sample size increases.

When the simulation was repeated with 500 circulating clones, power and VE estimates were nearly identical to those obtained when there were 10 circulating clones (Additional file 1: Figs. A2 and A3) for each scenario, estimator, and mean number of clones. This is because the mean number of clones is reduced by the same amount, regardless of which clones were transferred. There was only one difference from the previous set of simulations: with 500 circulating clones, VE_molFOI_ did not have higher power in Scenario 2 than in the other scenarios because there is no truncation on the number of clones vaccinees can experience. In general, communities with more circulating clones tend to have higher transmission. These two experiments produced similar results because, in the simulation model, the parameters driving differences in transmission levels are the exposure rate and the mean number of clones transferred per exposure, and these had the same values for the two simulations. For this reason, the simulations calibrated to each of the three analysed trials (which all assumed 50 circulating clones) capture differences in community transmission levels (by varying these two parameters) even if these communities actually have different numbers of circulating clones.

**Fig. 4:**
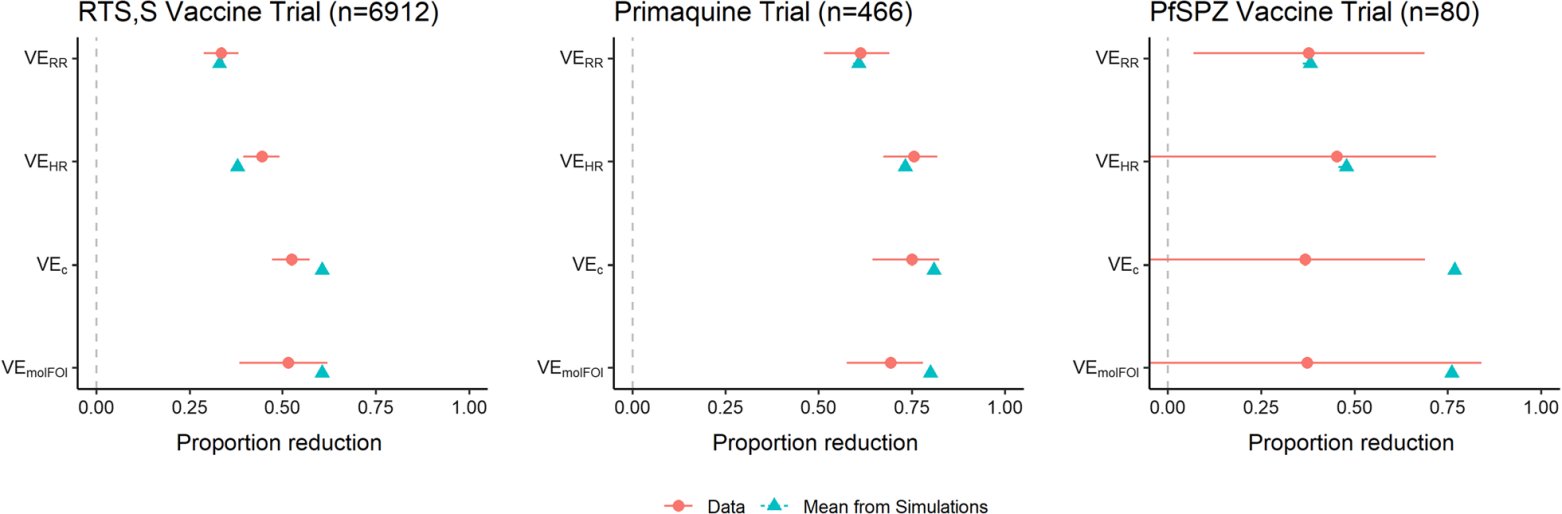
VE estimates (red circles) with 95% confidence intervals from three clinical trials. The blue triangles show expected values of the VE estimates from simulation models calibrated to match the event rates in both arms and the mean number of clones at first infection in controls

Figure 4 displays VE estimates and 95% CIs for the three trials analysed. Numeric values are in Additional file 1: Table A2, and Additional file 1: Table A3 provides summary statistics for each trial. The blue triangles show the average VE values induced by the simulation model that matches the event rates in each group and mean number of clones at first infection among controls. 95% confidence intervals for the mean VE from simulations are smaller than the plotting symbols. Note that these are different from the percentiles shown in Fig. 3, which displays variability between trials of the observed VE. The intervals in Fig. 4 display uncertainty in estimation of the true VE induced by the model (which can be made arbitrarily small by increasing the number of simulations). By design, the mean VE_RR_ from simulations is very close to the observed VE_RR_. The leftmost panel summarizes RTS,S trial results, which show that VE_HR_ is larger than VE_RR_, with a confidence interval farther from the null value of zero, and that VE_C_ and VE_molFOI_ are similar and are both higher than VE_HR_. VE_C_ has a confidence interval farther from the null value than VE_HR_, but VE_molFOI_ does not and has a wider interval. Information in this trial for VE_molFOI_ is limited because only the first infection was genotyped. The CIs are wider in the primaquine trial and much wider in the PfSPZ Vaccine trial due to the smaller sample sizes of these trials. In all three trials, the blue triangles show that the values of VE_C_ and VE_molFOI_ predicted by the simulation model are higher than those estimated from the data. Moreover, the model-predicted value of VE_C_ is significantly higher in RTS,S and PfSPZ (as the triangle lies to the right of the CI), but not the primaquine trial. The model-predicted value of VE_molFOI_ is significantly higher in the primaquine trial. The discrepancy between the simulation-predicted VE values and those from the data suggest that the simulation model tends to overestimate the VE measures incorporating genotyping data, which would cause it to overestimate the power gain from these approaches.

The following analyses were performed to explore possible reasons for the discrepancies between the simulation model results and estimates from the data:

*RTS,S Trial*: Summary statistics from the RTS,S data set were compared to those from simulations. Although the simulations match the event rates in each group, they predict a larger reduction in mean number of clones at first infection (from 2.26 in controls to 1.44 in vaccinees; while the actual vaccinees had a mean of 1.94 clones (95% CI [1.86, 2.02]) (Additional file 1: Table A4). The simulations also underestimate variability of the number of clones. The simulation was revised to match the entire distribution of clones in controls instead of using a truncated Poisson distribution that matches only the mean. This revision only slightly reduced the discrepancy between the data and the simulation summaries, so is not the source of the difference (Additional file 1: Table A4). Although the ordering of the RTS,S VE estimates is consistent with the simulation outputs (unlike the other estimates for the trials), the discrepancy between the data and the simulation summaries indicate that the simulations overestimate power for this data structure.

*Primaquine trial*: The treatment tested in this trial prevents reactivations of liver stage infections and is not expected to prevent new infections or reinfections from an outside source. Thus, the mechanism is different from that in the malaria vaccine simulation model. However, it is similar to the second scenario considered above—a vaccine that blocks half of all clones with 100% probability and no blocking for others. Although the set of clones to be blocked would differ between individuals in this trial, the model might be an adequate approximation.

The observed treatment effect in this trial on molFOI will eventually decline because after all liver-stage infections in controls have been reactivated, susceptibility to new *P. vivax* infections will be similar between controls and treated participants. The declining treatment effect is mathematically similar to a waning VE for a malaria vaccine, although the biological mechanism is different. Additional file 1: Fig. A4 shows monthly molFOI by treatment arm during follow-up. Control arm *P. vivax* molFOI surged in Months 2 and 3 post-enrollment, but the treated arm did not surge similarly. Hofmann et al*.* [16] conjectured that the surge could be due to a triggering of relapses from the blood-stage treatment or that relapses may have occurred soon after treatment (while anti-malarial drug levels were low), then were suppressed from detection as drug levels rose and were finally detected after drug levels waned. The molFOI for this group dropped steeply in Month 4 but remained larger than the molFOI in the treated arm for the rest of follow-up (Additional file 1: Fig. A4), possibly because reactivations continued at a slower rate among controls. This pattern is consistent with models of *P. vivax* recurrence in other studies [22–24]. Further modelling to understand the mechanisms influencing *P. vivax* reactivation is needed and is beyond the scope of this paper. The simulation model does not implement waning, and the power gain from the molecular force of infection approach is less when waning occurs because this measure incorporates information from all infections and does not incorporate the time to first infection.

VE_C_, however, is based on the time to first infection and number of clones present at first infection. The mean number of clones at first infection was 1.46 in controls and 1.49 in treated participants, making VE_C_ smaller than VE_HR_. When the analysis was repeated with only the first three months of follow-up, the genotyping VE estimates were larger than VE_HR_ with confidence intervals farther from the null value (Additional file 1: Table A2), but the mean numbers of clones at first infection were 1.51 and 1.44 in control and treated arms, which do not differ significantly or substantively. The simulation model induces a relationship between the risk reduction and the mean ratio, which is why the simulation-predicted value for VE_c_ is greater than that for VE_HR_. The combination of a dramatic risk reduction and null mean ratio observed in this trial are not what one expects from the model, but the model-predicted values of both VE_c_ and VE_HR_ are within the confidence intervals generated from this trial.

The simulations used exponential infection durations, which may result in too many short durations, giving too many opportunities for new infections. To test if this was causing the model to overestimate power, the original simulation study was revised assuming all infections lasted longer than the follow-up period. Results were similar (Additional file 1: Tables A5 and A6), so this assumption was not the root of the problem. Finally, a sensitivity analysis in which new post-baseline *P. vivax* infections with zero new *P. vivax* clones detected were imputed to have 1 new clone gave nearly identical results to the primary analysis (Additional file 1: Table A2).

*PfSPZ vaccine trial*: The small sample size of this trial creates a large amount of uncertainty in estimates. The mean number of clones at first infection was higher among vaccinees than controls (3.46 *vs*. 3.00), but the difference was not statistically significant (t-test p = 0.30). The ratio of mean number of clones at first infection for vaccinees to controls is 1.15. The RTS,S trial showed the opposite pattern: there were significantly fewer mean number of clones in vaccinees (1.94) *vs*. controls (2.26) at first infection. When 10,000 data sets of size 80 were resampled from the RTS,S data set, vaccinees had fewer clones than controls in only 71% of resampled data sets. They had more clones than controls in 29% of resamples, and the ratio of means was ≥ 1.15 in 14% of resampled daets. This means that 14% of trials of size 80 of a vaccine that reduces the number of clones at first infection as much as the RTS,S vaccine did will give a ratio of means ≥ 1.15. Therefore, it is difficult to draw a conclusion about what the PfSPZ Vaccine is doing, in terms of number of clones at first infection, from this trial.

## Simulation study 3

The third simulation study entailed resampling data sets of smaller sizes from the RTS,S data to estimate power with a realistic data structure and no model assumptions. The first three rows of Table 3 show that power estimated by this approach is modestly higher with VE_C_ than VE_HR_. The fourth row shows power estimated by imposing artificially higher event rates but preserving the time-to-event distributions among people who became infected as well as the distribution of number of clones. With these higher event rates and a higher risk ratio, power is lost rather than gained when genotyping data is added. This is because although the addition of genotyping data can add information, it also adds variability to the estimator because the two means are being estimated, so the confidence intervals are wider.

**Table 3:** Power estimated by resampling smaller trials from the RTS,S data

Control arm event rate	Vaccine arm event rate	Sample size	VE_HR_ power	VE_C_ power
0.38	0.26	80	0.29	0.33
0.38	0.26	150	0.52	0.56
0.38	0.26	250	0.75	0.78
0.60	0.30	80	0.85	0.80

The first three rows show estimated power from direct resamples of the RTS,S data. The fourth row shows power from an artificially imposed higher control group event rate and larger risk ratio reduction but matches the mean clones and time-to-even distributions among infected participants in each group. Because time-to-event distributions match only those among infected RTS,S participants but fewer people escape infection than in the RTS,S data, the hazard ratio for the fourth row does not match that in the RTS,S data

The increased uncertainty by adding genotyping information can be seen directly from the formula for the standard error of the estimator derived in [10]. The estimator is ${\widehat{\mathrm{VE}}}_{\mathrm{c}}= 1-\frac{{\overline{x} }_{v}}{{\overline{x} }_{c}}\mathrm{HR}=1-\widehat{\Delta }.$ As noted previously, the standard error for the estimator for log($\Delta$) is:

$${\mathrm{SE}}_{\mathrm{log}\widehat{\Delta }}= \sqrt{{{\mathrm{SE}}_{\widehat{\mathrm{logHR}}}}^{2}+\frac{{\mathrm{s}}_{\mathrm{v}}^{2}}{{\mathrm{I}}_{\mathrm{v}}{{\overline{\mathrm{x}} }_{\mathrm{v}}}^{2} }+\frac{{\mathrm{s}}_{\mathrm{c}}^{2}}{{\mathrm{I}}_{\mathrm{c}}{{\overline{\mathrm{x}} }_{\mathrm{c}}}^{2}}}$$

**Fig. 5:**
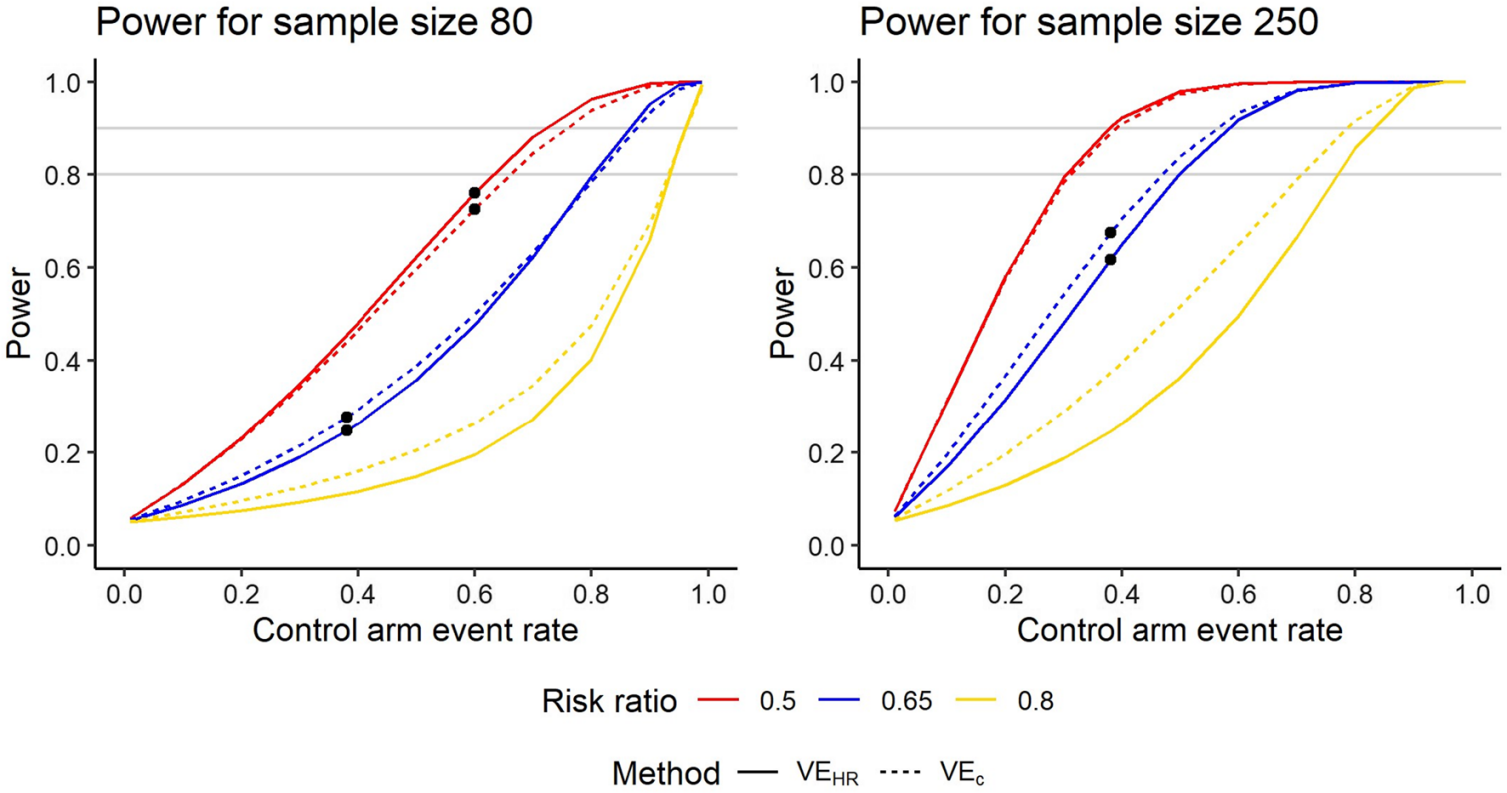
Power curves for sample sizes 80 and 250 for VE_HR_ and VE_C_. The calculations assume exponential time-to-event distributions, and the means and standard deviations of number of clones at first infection in each group match those in the RTS,S data. The black dots correspond to combinations of input parameters in Table 3. The analytic power estimates differ slightly from those in Table 3 because the RTS,S time-to-event distribution is not exponential, but they show the same general pattern

Thus, the variance of the estimator for log($\Delta$) is always greater than the variance of the log-transformed hazard ratio. Since 95% confidence intervals for the log-transformed estimands are calculated as Estimate ± 1.96 $\sqrt{\mathrm{Var}(\mathrm{Estimator})},$ the above variance formulas mean that the confidence interval for log(Δ) is always wider than that for log(HR). Adding genotyping information into the efficacy estimator can improve power when the reduction in mean clones is large and added variability is small, but can decrease power when the reduction in mean clones is small and added variability is large. Analytic formulas for power were derived to try to establish a threshold beyond which power is lost rather than gained by adding genotyping information (Additional file 1: Appendix, Additional file 1: Sect. 2). However, these formulas show that power depends on the sample size in each group, the numbers infected in each group, the hazard ratio, and the means and standard deviations of numbers of clones at first infection in each group. The threshold at which VE_C_ starts to have less rather than more power than VE_HR_ depends on all these parameters and cannot be reduced to a simple formula. Figure 5 shows power curves for sample sizes 80 and 250 for three different risk ratios. To reduce the number of input parameters for the power curves and create a simpler visualization, an exponential distribution was assumed for the time to first infection. In this case, the HR is defined by the infected proportions in each group as $\mathrm{HR}= \frac{\mathrm{log}(1-{p}_{v})}{\mathrm{log}(1-{p}_{c})}.$ The simulation results in Table 3 and power curves in Fig. 5 show that with a greater risk reduction, the extra information from the relatively small mean reduction observed in RTS,S is outweighed by the additional uncertainty added from estimating two means.

The derivations in the Additional file 1: Appendix show that the threshold reversing the power gain from VE_C_ does not depend on the sample size. The threshold occurs in the same place in the left and right graphs. For a risk ratio of 0.65, the threshold occurs at a control arm event rate of 0.74. The magnitude of the power difference between the two methods does depend on the sample size, however.

## Discussion

This paper compared two new malaria vaccine estimators incorporating genotyping data to the standard estimators by evaluating their operating characteristics in simulations and applying them to data from three trials. Both genotyping estimators were more powerful than standard estimators in model-based simulations, but analysis of the three trials suggests that the simulation model overestimates power. Despite this limitation, the simulation model gave insight into the performance of the two new VE methods. They control Type 1 error in all settings considered. In addition, the simulations showed that power from the genotyping methods generally did not differ when the vaccine blocked certain clones differentially as long as it blocked the same number of clones on average. An exception is a vaccine that completely blocks a subset of clones when there are a relatively small number of circulating clones, because this can reduce the variability of the estimator. The simulations also showed that the potential power gain increases with number of clones transferred per exposure, so there is less potential benefit in communities with few circulating clones, and VE_c_ showed no benefit with a single clone transferred per exposure. Communities with more circulating clones tend to have a higher exposure rate and more clones transferred per exposure. When these parameters are fixed, however, results are similar if the number of circulating clones increases.

The second simulation study entailed resampling smaller trials from the RTS,S vaccine trial. This does not rely on any model assumptions. This study showed that VE_C_ has moderately higher power than VE_HR_ in some realistic scenarios and lower power in others because there is a balancing act between adding data that can be informative about the vaccine and the additional variability in estimation contributed by estimating the two means. In data resembling the RTS,S trial, a power gain of 3 to 4 percentage points in small trials was found. The reduction in mean number of clones outweighs the increase in variability of the test statistic, and power is gained. When the risk reduction is larger than that seen in RTS,S, incorporating the mean reduction adds relatively more variability, and power is lost. In early phase trials, there is typically have a lot of uncertainty around the inputs going into the power calculation, so this estimator is not recommended as a primary efficacy approach.

There is less information in the three trials for understanding VE_molFOI_ than VE_C_ since only the primaquine trial included genotyping results from serially sampled blood, and this one was a treatment trial rather than a vaccine trial and so it has a different mechanism of action. VE_molFOI_ performed well in power simulations, but the treatment trial had a declining treatment effect for this endpoint, and the temporal decline decreases the size of the estimator and makes this method less powerful than VE_HR_. In the RTS,S trial, the value of VE_molFOI_ was higher than that of VE_HR_, but the confidence interval was much wider since only the first infection was genotyped. In RTS,S, the mean number of clones at first infection was significantly lower in vaccines than controls (1.94 vs. 2.26; p < 0.0001, t-test), unlike the other two trials. Furthermore, trials of RTS,S administered with AS02A or AS01B adjuvant also found the vaccine to reduce number of clones at first infection detected by microscopy on biweekly blood samples in children under five in Mozambique [25] and to reduce number of clones in all infections detected by weekly microscopy during a 3 month period in adults in western Kenya [26]. Depending on the extent of waning of the RTS,S vaccine in 12 months and the ratio of mean number of clones at each infection, it is possible that the VE_molFOI_ would be more powerful than VE_HR_ in trials with similar control arm event rate and vaccine mechanism but with genotyping results from serial samples. In the PfSPZ Vaccine trial analysed in this paper, vaccinees had more clones at first infection than controls (3.46 vs 3.00, p = 0.30), but the difference was not statistically significant, and simulations showed that this difference is a plausible value even if the true effect is actually the reduction observed in the much larger RTS,S trial. The sample size of the PfSPZ trial is too small to draw conclusions about VE_molFOI_. Genotyping measures from serially sampled blood from a much larger malaria vaccine trial would help determine if VE_molFOI_ may be helpful.

A major consideration of early phase endpoints is their performance in distinguishing which vaccines will be most likely to demonstrate an effect on the clinical endpoint of interest when they are tested in later phase trials. If a reduction in the number of clones does not also reduce the risk of clinical malaria, then adding genotyping information to VE estimators is just adding noise that could obscure the ability to distinguish performance between different vaccine candidates in early phase trials rather than adding information that will help differentiate their performance. Many individuals with polyclonal infections do not have symptomatic malaria, so vaccines which reduce their number of clones may have larger VE estimators by the genotyping approaches than other vaccines, even though they may not do as well at preventing clinical malaria. Therefore, even if VE_molFOI_ can increase the size of VE estimators for some vaccines, the risk of adopting this measure as a primary outcome should be carefully considered. It would be safer to include it as a secondary or exploratory outcome to better understand its relationship to clinical malaria as well as to the epidemiology and natural history of the disease. Such data could also help with understanding the mechanism of action of vaccines and treatments. Molecular data is often collected in malaria vaccine trials to test for specificity to certain parasite genotypes, but sampling schemes vary. The specification of genotyping VE endpoints as secondary or exploratory endpoints can help ensure the needed data will be collected.

A necessary condition for either of these genotyping endpoints to be a good surrogate is a positive relationship to clinical malaria. Molecular force of infection was positively related to clinical malaria in studies of infants and children [5, 16] and of a broader age population [27]. For VE_C_, a positive relationship would be needed between clinical malaria and the number of clones (also called the multiplicity of infection, MOI) at first infection, and evidence for this is mixed. The RTS,S trials cited earlier (including the one analysed here) support a positive relationship, as do some longitudinal studies of children [28, 29]. However, cross-sectional studies have found similar MOI between asymptomatic and symptomatic participants [30], and lower MOI in symptomatic than asymptomatic infections [31, 32]. This could be because the higher density of one dominating strain driving the infection makes it harder to detect the presence of other strains. It could also be due to the confounding effect of immunity: only immune individuals can have asymptomatic infections, and in a cross-sectional sample MOI will go up with increasing suppressive immunity (lower immunity will lead to clinical symptoms with the first infecting strain). For this reason, results from cross-sectional studies are difficult to interpret. A longitudinal analysis of pooled individual data found baseline MOI in asymptomatic people to be related to subsequent clinical malaria in children under 5, but that the relationship varied by transmission setting for children over 5 [33] and was negative for children over 5 in high transmission settings. Baseline MOI in asymptomatic people is likely related to MOI of the first infection during follow-up because multiple clones can be transferred per bite [18] and asymptomatic people with higher baseline MOI may have higher exposure. Therefore, potential surrogacy of VE_C_ may depend on the age of the population and the transmission setting. It is also unclear how this would differ from vaccine clinical trials where antimalarial treatment is frequently used at the beginning of the observation period, creating a situation where each clone detected represents a new infection. An association with clinical malaria is a necessary but not sufficient condition for a good surrogate outcome. A surrogate must also lie in the disease process pathway on which the intervention acts and capture all relevant on-target and off-target effects of the intervention on the disease process [1]. Generally, this comprehensive understanding of the disease process is impossible, and surrogacy is instead validated by a meta-analysis relating the treatment effect on the surrogate to the treatment effect on the clinical outcome in multiple trials [34]. Although this validation is not typically required for early phase trial endpoints, these issues are discussed here to point out the risk of substituting one early-phase trial endpoint with another.

Although infection detected by microscopy is a standard early phase outcome, its relationship to clinical malaria as a surrogate is also unclear since the development of disease from blood-stage infection depends on immunity and exposure, which differ between individuals but tend to be correlated since past exposure is a predictor for current immunity. For example, a vaccine that blocks blood stage infections only in the most robust individuals (who would not have developed clinical malaria anyway), could reduce malaria infection but not reduce clinical malaria in individuals. Genotyping data can help explain these relationships. For example, one study found that antibody levels were associated with increased risk of clinical malaria in children aged 1–4 in Papua New Guinea, but adjustment for molFOI removed most of the association, indicating that the antibody levels were a proxy for exposure [35]. When the analysis was repeated on a cohort aged 5–14 years, antibody levels were associated with protection from clinical malaria, suggesting that a threshold level of antibody levels is needed to reduce the risk of disease. Collection and analysis of genotyping data on a large scale is needed to further disentangle these relationships [36].

Even if they are not good surrogates for clinical endpoints, genotyping VE endpoints may serve other purposes. A vaccine which reduces the rate of acquisition of new clones but not clinical malaria (for example, by reducing molFOI only in robust people who would not have become ill) can reduce the malaria caseload in a community and therefore have a beneficial community-level effect. Regulatory approval generally requires an endpoint representing how a person “feels, functions, or survives” or a validated surrogate of such an endpoint. This requirement helps ensure that the direct individual benefit outweighs the risks of the intervention. An intervention with a community benefit but no direct individual benefit to recipients may need a different approval pathway [37]. Such a pathway and the use of alternative Phase 3 endpoints would increase efficiency of Phase 3 trials of interventions in malaria-experienced populations whose clinical malaria rate is too low to provide adequate power for feasible sample sizes.

This study has limitations. Although the simulation model gave insight into the performance of the two new estimators, it overestimated the increase in the VE value for the VE methods incorporating genotyping data. Thus, it may also overestimate the power gain from these methods. The model-based simulations calibrated to the RTS,S trial estimated a larger reduction in number of clones at first infection than what was observed. The simulation model assumed a purely leaky vaccine, meaning that the vaccine reduces the probability of infection equally for all vaccinated participants. The RTS,S vaccine may instead be a combination of all-or-nothing and leaky. Models have been developed to test whether a vaccine is all-or-nothing, leaky, or combination, but require genotyping data from multiple malaria exposures [38], which were not available in this data set. Another possible explanation for the discrepancy is that the model assumed equal distributions of the clones in circulation, perfect sensitivity in detecting all clones, and no competition between clones. It is possible that in infections with larger numbers of clones (e.g., in controls), the clones present in lower frequencies may be harder to detect. “Competitive release”, in which clearance of some clones creates space for others to flourish [39], could also water down the vaccine-induced reduction in number of clones. The observed 10–15% missing genotyping results in the three trial data sets analysed indicates imperfect sensitivity, which could explain some of the discrepancy. The simulation model did not include antimalarial treatment blackout periods (removal of time not-at-risk during antimalarial treatment). For an effective vaccine, more controls will become ill than vaccinees, so subtracting blackout periods would reduce the time-at-risk (the denominator for molFOI) more for controls than for vaccinees, inflating the treatment effect estimates. However, this approach is consistent with the analysis of the trials, and in the RTS,S and PfSPZ Vaccine trials, antimalarial medication was likely not given before the first detected malaria infection.

Another limitation is that two of the three trials measured genotyping data only at the first observed infection by thick blood smear microscopy. Only the primaquine trial followed the optimal sampling scheme for the proposed VE estimators: PCR testing of serially sampled blood with genotyping performed on all PCR positive samples. Because microscopy is less sensitive than PCR, some first infections may have been missed in the PfSPZ Vaccine trial. The RTS,S trial used clinical malaria as a trigger for both microscopy and genotyping analysis, so asymptomatic first infections would have been missed. The trial’s population was infants, who generally have fewer asymptomatic infections than older participants. Since genotyping was not performed on repeated samples, power for VE_molFOI_ was limited in these two trials, as was information about the molFOI time trend.

A third limitation is that the primaquine trial was not a vaccine trial, but these genotyping efficacy estimators could be useful for trials of any malaria prevention intervention. The primaquine treatment mechanism differed from our simulation model, but it resembles the simulation scenario in which some clones were always blocked. The deviations between model-predicted and data-estimated statistical summaries were similar between the three trials, suggesting that the operating characteristics of the estimators are similar for this type of treatment trial and for vaccine trials. Since the primaquine treatment was completed before the follow-up period began, waning was inevitable after all sequestered *P. vivax* infections among controls were reactivated. The similar (rather than improved) performance of VE_molFOI_ and VE_C_ in this trial compared to the standard estimators was due in part to waning. This performance can be expected for any intervention with waning, including vaccines in development.

## Conclusion

This is the first paper to explore the potential benefit of incorporating genotyping data into VE measures for early phase trials by systematically studying their operating characteristics in a range of settings and vaccine mechanisms and applying them to three clinical trials. The analyses suggest that VE_C_ is not likely to systematically improve upon the widely-used VE_HR_ in early phase malaria vaccine trials. VE_molFOI_ was most powerful in simulations, but data analyses suggest that it may not improve power in trials with waning VE. Because there were limited data available to evaluate this measure, it merits further exploration, which would require genotyping data from serially sampled blood for one or more large malaria vaccine trials. Such data would be used to explore multiple scientific questions [40] and to determine the scenarios in which VE_molFOI_ will be most useful and informative.

### Supplementary Information

**Additional file 1: Table A1.** Input parameters for Simulation Study 1, comparing VE methods for different vaccine-blocking scenarios and mean number of clones transferred per exposure. **Figure A1.** Simulation-estimated power by vaccine blocking mechanism, number of clones transferred per exposure, and VE method, assuming 10 clones circulating in the community. **Figure A2.** Simulation-estimated power by vaccine blocking mechanism, number of clones transferred per exposure, and VE method, assuming 500 clones circulating in the community. **Figure A3.** Estimated power, assuming 500 circulating clones, by the four different VE methods by mean number of clones transferred per exposure (Panel A) and mean VE estimator values across simulations (Panel B) with variability represented by 2.5^th^ and 97.5^th^ percentiles. **Table A2.** Efficacy estimates for the three trials analyzed. **Table A3.** Summary statistics for the three trials analyzed. **Table A4.** Summary statistics from simulations calibrated to RTS,S trial data. **Figure A4.** Monthly molecular force of *P. vivax* blood stage infection (molFOI) in the two treatment arms in the primaquine trial. **Table A5.** Simulation results with exponential durations (mean of 6 months for females; 10 months for males). **Table A6.** Simulation results with durations longer than follow-up period (all durations = 200 days for a 168 day follow-up). **Figure A5.** Analytic and simulation-estimated power curves for VE_HR_ and VE_C_ for trials with data structure matching the RTS,S data, assuming exponential time-to-event distributions.

## Data Availability

RTS,S Data: The authors do not have permission to share this data set, but the results in this manuscript are consistent with previously published analyses [12]. The Additional file for that publication includes code and a fake data set (with the same structure as the real data set) so that readers can test the statistical methods on the fake data set [12]. PfSPZ Data: The data are publicly available here: https://osf.io/bfamc/. Primaquine Data: The data are publicly available [17].
