# Supplementary material for: Can incorporating genotyping data into efficacy estimators improve efficiency of early phase malaria vaccine trials?
Source: Malar J. 2023 Dec 19;22:383. doi: 10.1186/s12936-023-04802-0 (PMC10729369; doi:10.1186/s12936-023-04802-0)
Supplement: Supplementary file 1 — Additional file 1: Table A1. Input parameters for Simulation Study 1, comparing VE methods for different vaccine-blocking scenarios and mean number of clones transferred per exposure. Figure A1. Simulation-estimated power by vaccine blocking mechanism, number of clones transferred per exposure, and VE method, assuming 10 clones circulating in the community. Figure A2. Simulation-estimated power by vaccine blocking mechanism, number of clones transferred per exposure, and VE method, assuming 500 clones circulating in the community. Figure A3. Estimated power, assuming 500 circulating clones, by the four different VE methods by mean number of clones transferred per exposure (Panel A) and mean VE estimator values across simulations (Panel B) with variability represented by 2.5th and 97.5th percentiles. Table A2. Efficacy estimates for the three trials analyzed. Table A3. Summary statistics for the three trials analyzed. Table A4. Summary statistics from simulations calibrated to RTS,S trial data. Figure A4. Monthly molecular force of P. vivax blood stage infection (molFOI) in the two treatment arms in the primaquine trial. Table A5. Simulation results with exponential durations (mean of 6 months for females; 10 months for males). Table A6. Simulation results with durations longer than follow-up period (all durations = 200 days for a 168 day follow-up). Figure A5. Analytic and simulation-estimated power curves for VEHR and VEC for trials with data structure matching the RTS,S data, assuming exponential time-to-event distributions. [file 12936_2023_4802_MOESM1_ESM.docx]

**Additional File 1: Supplementary Appendix to**

**“Can incorporating genotyping data into efficacy estimators improve efficiency of early phase malaria vaccine trials?”**

Gail E. Potter, Viviane Callier, Biraj Shrestha, Sudhaunshu Joshi, Ankit Dwivedi, Joana C. Silva, Matthew B. Laurens, Dean A. Follmann, Gregory A. Deye

Contents

[Supplementary Methods Section 1: Technical Details for Simulation Study 1 2](#_Toc151129820)

[Table A1. Input parameters for Simulation Study 1, comparing VE methods for different vaccine-blocking scenarios and mean number of clones transferred per exposure. 3](#_Toc151129821)

[Figure A1: Simulation-estimated power by vaccine blocking mechanism, number of clones transferred per exposure, and VE method, assuming **10 clones** circulating in the community. 4](#_Toc151129822)

[Figure A2: Simulation-estimated power by vaccine blocking mechanism, number of clones transferred per exposure, and VE method, assuming **500 clones** circulating in the community. 5](#_Toc151129823)

[Figure A3. Estimated power, assuming **500 circulating clones**, by the four different VE methods by mean number of clones transferred per exposure (Panel A) and mean VE estimator values across simulations (Panel B) with variability represented by 2.5^th^ and 97.5^th^ percentiles. 6](#_Toc151129824)

[Table A2: Efficacy estimates for the three trials analyzed. 7](#_Toc151129825)

[Table A3: Summary statistics for the three trials analyzed. 7](#_Toc151129826)

[Table A4. Summary statistics from simulations calibrated to RTS,S trial data 8](#_Toc151129827)

[Figure A4: Monthly molecular force of *P. vivax* blood stage infection (molFOI) in the two treatment arms in the primaquine trial. 9](#_Toc151129828)

[Table A5: Simulation results with exponential durations (mean of 6 months for females; 10 months for males) 10](#_Toc151129829)

[Table A6: Simulation results with durations longer than follow-up period (all durations = 200 days for a 168 day follow-up) 10](#_Toc151129830)

[Supplementary Methods Section 2: Analytic power derivations 11](#_Toc151129831)

[Figure A5. Analytic and simulation-estimated power curves for VE_HR_ and VE_C_ for trials with data structure matching the RTS,S data, assuming exponential time-to-event distributions. 14](#_Toc151129832)

[REFERENCES 15](#_Toc151129833)

Supplementary Methods Section 1: Technical Details for Simulation Study 1

To compare power of the different approaches, we performed a simulation study exploring a range of parameters. The simulation worked as follows:

1. Half of the n participants were randomized to the vaccine arm and half to placebo.
2. Exposure events were simulated with equal rates in the two arms via a Poisson process. We define an “exposure event” as exposure sufficient to cause a blood-stage infection in a control participant, so the event may correspond to multiple infectious bites.
   1. We assumed an average of 120 days between events so that 75% of participants (1-exp(-168/120) experience at least one exposure during a 168-day (6-month) follow-up period.
3. Each exposure event may transfer multiple *P. falciparum* clones, which then may be blocked by the vaccine. For each exposure event, the number of clones transferred (n_c_) was sampled from a truncated Poisson distribution with mean 3. The truncation forces values to range from 1 to 10 since we assumed 10 clones are circulating in the community. Then, the specific n_c_ clones transferred during the bite were sampled from the set of 10 circulating clones.
4. Vaccine blocking was simulated according to different scenarios:
   1. Scenario 1: To simulate performance of a vaccine that works identically for all clones, each clone was blocked with probability 0.5. This means that for vaccinees, each clone present in a bite is half as likely to lead to a blood stage infection than for controls. The infection data for each vaccinee includes only the non-blocked clones.
   2. Scenario 2: To simulate performance of a vaccine that protects perfectly against a subset of clones (set A), but confers no protection against others (Set B), clones from Set A are removed while none from Set B are removed. We assumed that half of the circulating clones are in Set A and half in Set B.
   3. Scenario 3: To simulate performance of a vaccine that protects partially (75%) against clones in set A and confers lower protection (25%) against those in Set B, clones from Set A are removed with probability 0.75 and those from Set B are removed with probability 0.25.
   4. Scenario 4: To confirm that Type 1 error is controlled, an ineffective vaccine was simulated.
5. For each clone, the infection duration was sampled from exponential distributions with a mean of 303 days for females and 167 days for males (Means are from Briggs et al. [1]). Enrollment was assumed to be equal between the sexes.
6. An “observed” data set was created by sampling from the simulated data at 14-day intervals over a 168-day follow-up period. The thick blood smear test is assumed to have perfect sensitivity and specificity. We assumed no dropout.
7. Estimates for the different endpoints/estimands were calculated with p-values as follows:
   1. Risk ratio approach: Fisher's exact test was used to test for a difference in proportions.
   2. Hazard ratio approach: Cox regression was performed to calculate the hazard ratio based on the time to first positive TBS results. People not infected during follow-up were censored at day 168. A p-value was calculated with the score test.
   3. molFOI approach: For each person, the number of unique clones present in the first positive TBS were counted. In subsequent samples, new clones were added to the count, and repeat clones were added if separated from their prior occurrence by a TBS showing the absence of that clone. Time-at-risk was set to 168 days for all people. Quasi-poisson regression was performed with log-transformed time-at-risk as an offset and a treatment group indicator to estimate the incidence rate ratio.
   4. VEc approach: Let $\hat{\beta}$ denote the log HR from the Cox model in (a), Z be a treatment group indicator, and let $\bar{X}_{Z}$ denote the mean of clones for the first infection among vaccinees (Z=1) and controls (Z=0). We calculated VE_C_ as 1 – $\hat{\Delta}$, where Δ was estimated by $\hat{\Delta}=$exp($\hat{\beta}$) $\bar{x}_{1}$/$x_{0}$_._  We performed a Wald test comparing log(Δ) to the null value of zero since log($\hat{\Delta}$) rather than $\hat{\Delta}$ is asymptotically normal. The standard error for $log(\hat{\Delta})$ was previously derived using the delta method [2]. Letting I_Z_ denote the number infected in group Z and $s_{Z}^{2}$ denote the variance of the number of clones of the first infection in group Z, the standard error of $log(\hat{\Delta})$ is:

$${SE}_{log\hat{\Delta}}= \sqrt{{{SE}_{\hat{\beta}}}^{2}+\frac{s_{1}^{2}}{I_{1}{x_{1}}^{2}}+\frac{s_{0}^{2}}{I_{0}{\bar{x}_{0}}^{2}}}$$

The Wald statistic was calculated as $\frac{log(\hat{\Delta})}{{SE}_{log\hat{\Delta}}}$, and the null hypothesis was rejected when this statistic was < -1.96 or >1.96.

1. 1000 simulations were performed for each combination of input parameters (Table A1). Power was estimated for each endpoint as the proportion of simulations for which the null hypothesis was rejected.

Table A1. Input parameters for Simulation Study 1, comparing VE methods for different vaccine-blocking scenarios and mean number of clones transferred per exposure.

| Parameter | Input values |
| --- | --- |
| Sample size | 60, 80, 100, 120, 140 |
| Follow-up duration | 6 months (168 days) |
| Proportion of participants infected at least once during follow-up | 75% |
| Average number of clones transferred per exposure | 1, 2, 3 |
| Number of clones in community | 10 |
| Vaccine blocking scenario | 1: Block each clone with probability 50%  2: Block half of clones perfectly  3: Block half of clones with probably 0.75 and half with probability 0.25  4: No blocking |
| Infection duration | Exponential distributions with mean duration 303 days for males and 167 days for females. |
| Sampling scheme | Biweekly blood draws |

Figure A1: Simulation-estimated power by vaccine blocking mechanism, number of clones transferred per exposure, and VE method, assuming **10 clones** circulating in the community.


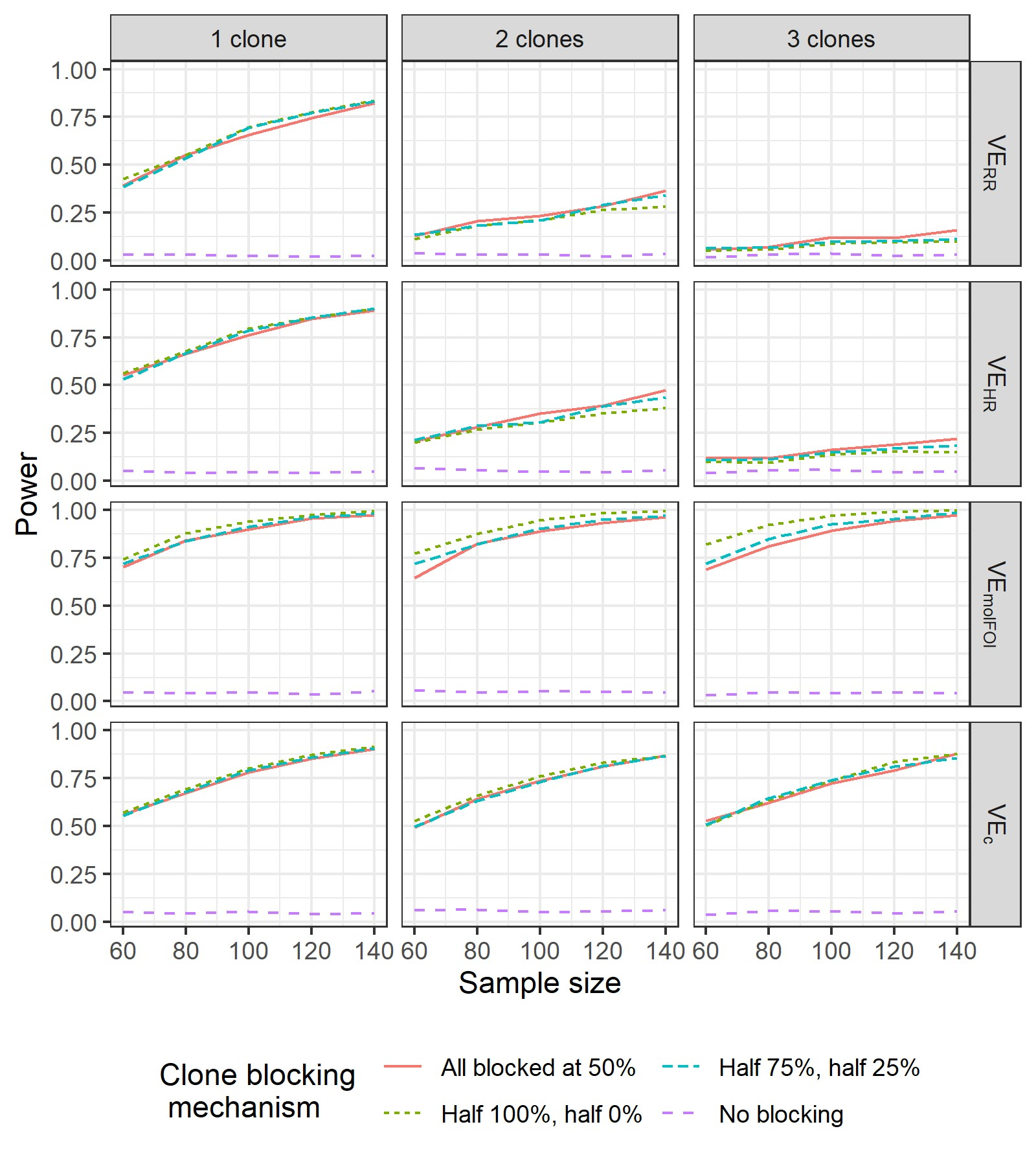


Figure A2: Simulation-estimated power by vaccine blocking mechanism, number of clones transferred per exposure, and VE method, assuming **500 clones** circulating in the community.


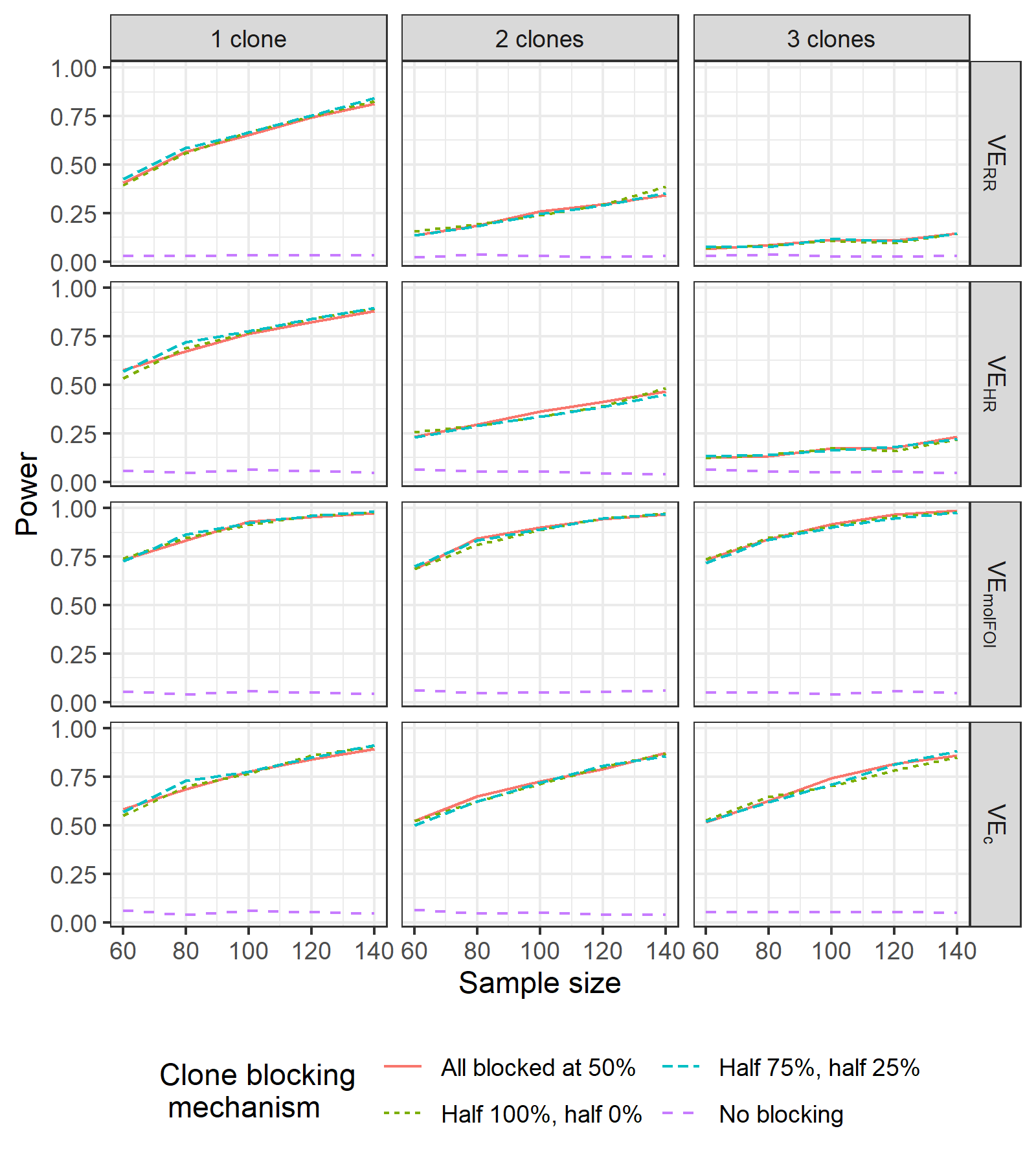


Figure A3. Estimated power, assuming **500 circulating clones**, by the four different VE methods by mean number of clones transferred per exposure (Panel A) and mean VE estimator values across simulations (Panel B) with variability represented by 2.5^th^ and 97.5^th^ percentiles.

VE methods were calculated for the same sample sizes; the displayed intervals are staggered to distinguish them visually. While the main text figure shows results for 10 clones circulating in the community, this figure shows results for 500 circulating clones and is nearly identical. In both sets of simulations, a maximum of 10 clones were transferred per exposure. Community transmission is driven by the exposure rate and the mean number of clones per exposure.


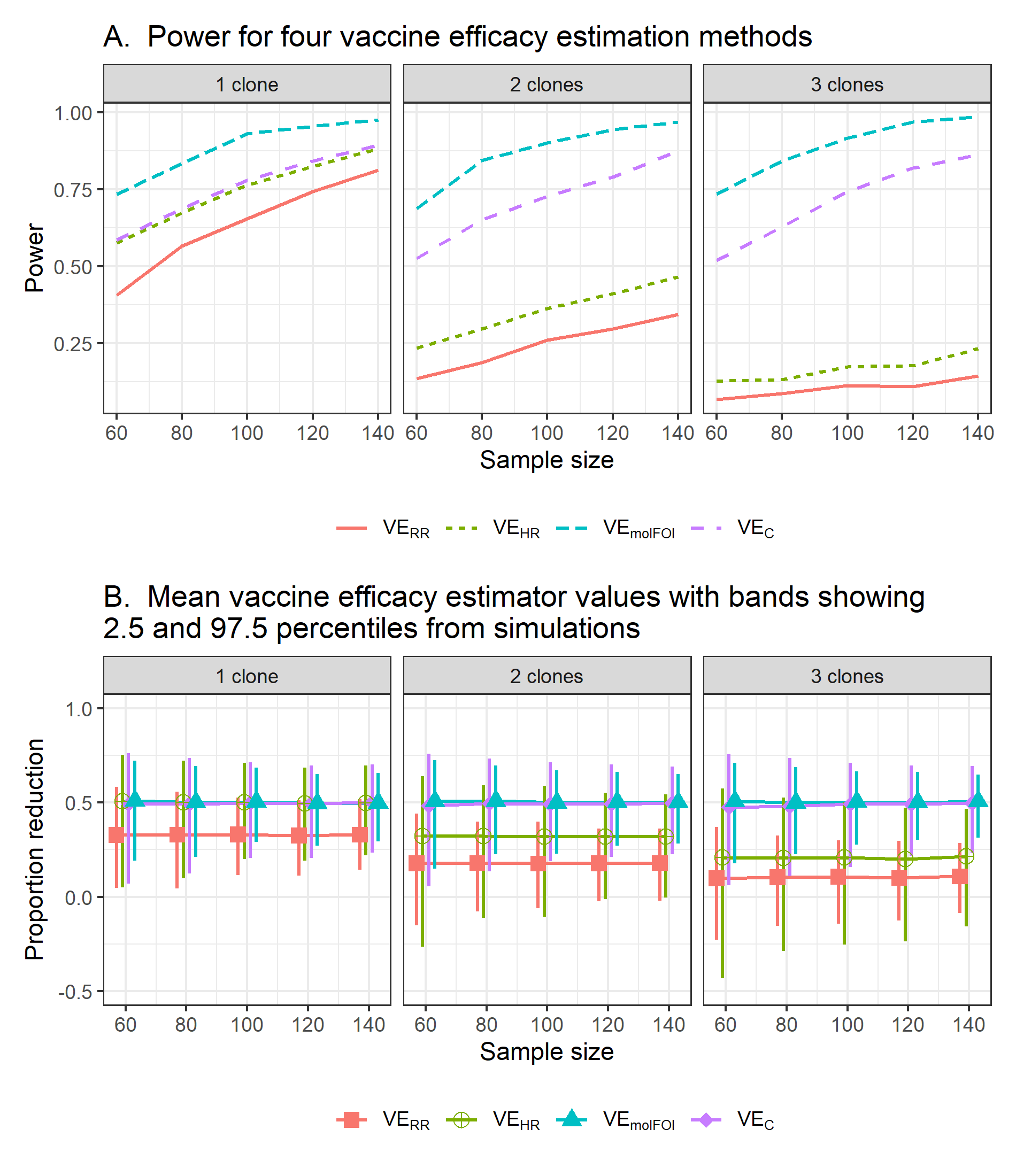


Table A2: Efficacy estimates for the three trials analyzed.

| **Method** | | **Estimate** | | **95% CI** | | **P-value** |
| --- | --- | --- | --- | --- | --- | --- |
| **RTS,S Vaccine Trial** | | | | | | |
| VE_RR_ = 1-RR | | 0.336 | | (0.288, 0.382) | | <0.001 |
| VE_HR_ = 1-HR | | 0.446 | | (0.395, 0.492) | | <0.001 |
| VE_C_ = 1-HR×($\bar{x}_{v}/\bar{x}_{c}$) | | 0.525 | | (0.472, 0.572) | | <0.001 |
| VE_molFOI_ = 1-$\mathrm{molFOI}_{v}/\mathrm{molFOI}_{c}$ | | 0.516 | | (0.385, 0.620) | | <0.001 |
| **Primaquine Trial** | | | | | | |
| VE_RR_ = 1-RR | 0.612 | | (0.515, 0.690) | | <0.001 | |
| VE_HR_ = 1-HR | 0.756 | | (0.673, 0.818) | | <0.001 | |
| VE_C_ = 1-HR×($\bar{x}_{v}/\bar{x}_{c}$) | 0.750 | | (0.644, 0.824) | | <0.001 | |
| VE_molFOI_ = 1-$\mathrm{molFOI}_{v}/\mathrm{molFOI}_{c}$ | 0.694 | | (0.576, 0.779) | | <0.001 | |
| **Primaquine Trial, sensitivity analysis** | | | | | | |
| VE_RR_ = 1-RR | 0.612 | | (0.515, 0.690) | | <0.001 | |
| VE_HR_ = 1-HR | 0.756 | | (0.673, 0.818) | | <0.001 | |
| VE_C_ = 1-HR×($\bar{x}_{v}/\bar{x}_{c}$) | 0.749 | | (0.649, 0.820) | | <0.001 | |
| VE_molFOI_ = 1-$\mathrm{molFOI}_{v}/\mathrm{molFOI}_{c}$ | 0.690 | | (0.575, 0.774) | | <0.001 | |
| **Primaquine Trial, first three months only** | | | | | | |
| VE_RR_ = 1-RR | 0.697 | | (0.596, 0.773) | | <0.001 | |
| VE_HR_ = 1-HR | 0.784 | | (0.695, 0.846) | | <0.001 | |
| VE_C_ = 1-HR×($\bar{x}_{v}/\bar{x}_{c}$) | 0.793 | | (0.684, 0.864) | | <0.001 | |
| VE_molFOI_ = 1-$\mathrm{molFOI}_{v}/\mathrm{molFOI}_{c}$ | 0.802 | | (0.708, 0.866) | | <0.001 | |
| **PfSPZ Vaccine Trial** | | | | | | |
| VE_RR_ = 1-RR | 0.377 | | (0.068, 0.687) | | 0.017 | |
| VE_HR_ = 1-HR | 0.452 | | (-0.066, 0.718) | | 0.076 | |
| VE_C_ = 1-HR×($\bar{x}_{v}/\bar{x}_{c}$) | 0.368 | | (-0.285, 0.689) | | 0.205 | |
| VE_molFOI_ = 1-$\mathrm{molFOI}_{v}/\mathrm{molFOI}_{c}$ | 0.373 | | (-1.458, 0.840) | | 0.505 | |

Table A3: Summary statistics for the three trials analyzed.

|  | RTS,S   (n=6912) | | Primaquine   (n=466) | | PfSPZ   (n=80) | |
| --- | --- | --- | --- | --- | --- | --- |
|  | Control | Treated | Control | Treated | Control | Treated |
| Number analyzed | 2335 | 4577 | 233 | 233 | 41 | 39 |
| Number infected during follow-up | 908 | 1181 | 165 | 64 | 23 | 14 |
| Percent infected during follow-up | 39% | 26% | 71% | 27% | 56% | 36% |
| Mean clones at first infection | 2.26 | 1.94 | 1.46 | 1.49 | 3.00 | 3.46 |
| SD clones at first infection | 1.55 | 1.33 | 1.03 | 1.01 | 1.03 | 1.33 |
| molFOI (new clones per year) | 1.34 | 0.65 | 6.08 | 1.86 | 5.26 | 3.30 |

Table A4 shows results from simulations calibrated to the RTS,S data by using the parameter inputs in Table 1 and a sample size of 500. The simulation model produced a bigger reduction in mean number of clones (2.26 to 1.42) than that observed in the RTS,S data (2.26 to 1.94 [95% CI: 1.86-2.02]), indicating that our model is not correct. The simulations also show less variability of number of clones in both groups and a hazard ratio closer to the null value. We updated the simulations to increase the variability of the number of clones. Instead of assuming that the number of clones transferred per exposure has a truncated Poisson distribution with mean=2.26, we sampled directly from the observed distribution of number of clones in the control group. Results are in column 3. With this update, our simulation model still underestimates variability in the control arm and overestimates the reduction in mean clones, both of which would lead us to overestimate power.

Table A4. Summary statistics from simulations calibrated to RTS,S trial data

| **Parameter** | **RTS,S data** | **Simulations** | **Simulations, RTS,S variance** |
| --- | --- | --- | --- |
| Hazard ratio (HR) | 0.55 | 0.62 | 0.61 |
| Probability of infection in controls | 0.39 | 0.38 | 0.38 |
| Probability of infection in vaccinees | 0.26 | 0.26 | 0.25 |
| Mean # clones at first infection for controls | 2.26 | 2.24 | 2.24 |
| Mean # clones at first infection for vaccinees | 1.94 [1.86, 2.02] | 1.42 | 1.47 |
| SD # clones at first infection for controls | 1.55 | 1.24 | 1.53 |
| SD # clones at first infection for vaccinees | 1.33 [1.24, 1.44] | 0.69 | 0.8 |

Figure A4: Monthly molecular force of *P. vivax* blood stage infection (molFOI) in the two treatment arms in the primaquine trial.

Treatment was given over a 28-day period before the beginning of the 8-month follow-up period and included chloroquine (CQ) for the first three days, primaquine (PQ) or placebo for 5 days per week, and artemether lumefantrine (AL) for Days 11-13. Separate VE_molFOI_ estimates for Months 1-3 and Months 4-8 show that the molFOI in the two treatment arms become closer together over the study period, causing the estimated treatment effect to decline over time.


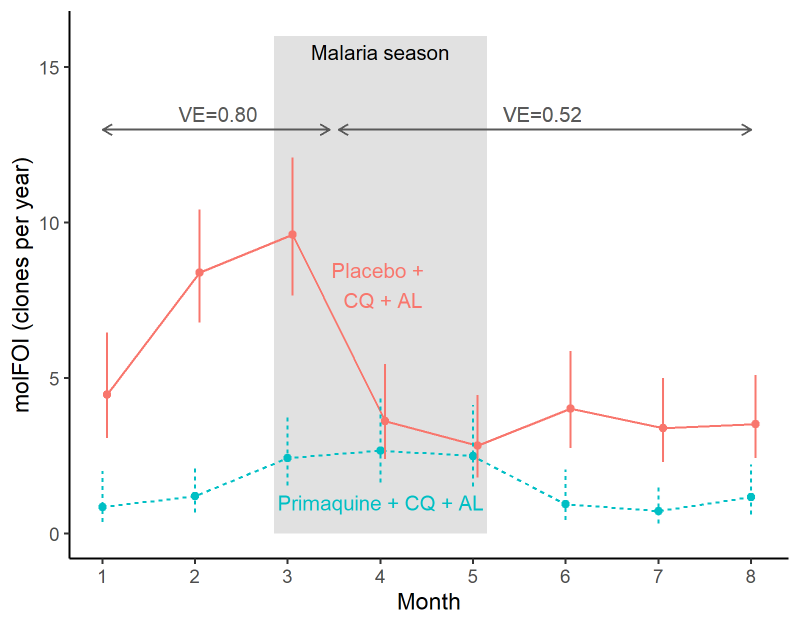


**Simulations with longer infection durations**

To assess whether our exponential infection duration distribution in simulations resulted in too many short durations, we redid the simulations forcing every infection with a new clone to last for more than the follow-up period. This is an extreme scenario to see how much our results would differ with no repeat infection. They did not differ much. Table 3 shows results from our first set of simulations, while Table 4 shows results from the same simulation with no repeat infection. We get more power with the longer durations since the infection durations in the original simulations are sometimes shorter than the sampling interval (14 days) so we miss some infections that way. Such short durations may be unrealistic.

Table A5: Simulation results with exponential durations (mean of 6 months for females; 10 months for males)

| #Clones controls | #clones vaccinees | P(infected)   controls | P(infected)   vaccinees | VE_RR_   power | HR | VE_HR_ power | #events   controls | #events   vaccinees | Ratio molFOI | VE_molFOI_ power | HR$\frac{\bar{x}_{v}}{\bar{x}_{c}}$ | VE_C_ power |
| --- | --- | --- | --- | --- | --- | --- | --- | --- | --- | --- | --- | --- |
| 1 | 0.5 | 0.74 | 0.5 | 0.52 | 0.51 | 0.65 | 1.28 | 0.67 | 0.52 | 0.8 | 0.52 | 0.66 |
| 2 | 0.99 | 0.75 | 0.61 | 0.17 | 0.68 | 0.26 | 2.44 | 1.29 | 0.52 | 0.82 | 0.51 | 0.63 |
| 3 | 1.5 | 0.75 | 0.68 | 0.07 | 0.8 | 0.14 | 3.45 | 1.89 | 0.55 | 0.83 | 0.52 | 0.62 |

Table A6: Simulation results with durations longer than follow-up period (all durations = 200 days for a 168 day follow-up)

| #Clones controls | #clones vaccinees | P(infected)   controls | P(infected)   vaccinees | VE_RR_  power | HR | VE_HR_ power | #events   controls | #events  vaccinees | Ratio molFOI | VE_molFOI_ power | HR$\frac{\bar{x}_{v}}{\bar{x}_{c}}$ | VE_C_ power |
| --- | --- | --- | --- | --- | --- | --- | --- | --- | --- | --- | --- | --- |
| 1 | 0.5 | 0.76 | 0.5 | 0.59 | 0.49 | 0.71 | 1.32 | 0.68 | 0.51 | 0.86 | 0.5 | 0.71 |
| 2 | 1 | 0.76 | 0.62 | 0.22 | 0.68 | 0.32 | 2.47 | 1.3 | 0.52 | 0.83 | 0.51 | 0.65 |
| 2.99 | 1.5 | 0.75 | 0.68 | 0.07 | 0.82 | 0.12 | 3.41 | 1.9 | 0.55 | 0.81 | 0.53 | 0.61 |

Supplementary Methods Section 2: Analytic power derivations

Notation:

- HR = hazard ratio
- logHR = log hazard ratio
- $p_{0}$= control group infection proportion
- $p_{1}$= vaccine group infection proportion
- $RR= p_{1}/p_{0}$ (risk ratio)
- $\mu_{0}$= control group mean # clones at first infection
- $\mu_{1}$= vaccine group mean # clones at first infection
- $\sigma_{0}^{2}=$ control group variance of # clones at first infection
- $\sigma_{1}^{2}=$ vaccine group variance of # clones at first infection
- n = sample size

Power for a one-sided test at 0.025 is the same as power for a two-sided test at 0.05 if the treatment effect is sufficiently large (since all rejections of the null hypothesis will be in only one direction). For simplicity the formulas below are for the one-sided test and assume an equal allocation ratio. For a negative outcome (e.g., malaria illness), a negative logHR is beneficial. Then, asymptotic power for a 1-sided test for VE by HR at 0.025 is [3]:

$$\Phi\left( -1.96-\frac{logHR}{\sqrt{\left( \frac{1}{(\frac{n}{2})p_{0}}+\frac{1}{(\frac{n}{2})p_{1}} \right)}} \right)=\Phi\left( -1.96-\sqrt{\frac{n}{2}}\frac{logHR}{\sqrt{\left( \frac{1}{p_{0}}+\frac{1}{p_{1}} \right)}} \right)=$$

$$\Phi\left( -1.96-\sqrt{\frac{n}{2}}\frac{logHR}{\sqrt{\left( \frac{1}{p_{0}}+\frac{1}{RRp_{0}} \right)}} \right)=\Phi\left( -1.96-\sqrt{\frac{np_{0}}{2}}\frac{logHR}{\sqrt{\left( 1+\frac{1}{RR} \right)}} \right)$$

Asymptotic power for a 1-sided test for VE by Follmann and Huang at 0.025 is:

$$\Phi\left( -1.96-\frac{logHR+log\frac{\mu_{1}}{\mu_{0}}}{\sqrt{\left( \frac{1}{(\frac{n}{2})p_{0}}+\frac{1}{(\frac{n}{2})p_{1}}+\frac{\sigma_{1}^{2}}{{\mu_{1}}^{2}(\frac{n}{2})p_{1}}+\frac{\sigma_{0}^{2}}{{\mu_{0}}^{2}(\frac{n}{2})p_{0}} \right)}} \right)=\Phi\left( -1.96-\sqrt{\frac{np_{0}}{2}}\frac{logHR+log\frac{\mu_{1}}{\mu_{0}}}{\sqrt{\left( 1+\frac{1}{RR}+\frac{\sigma_{1}^{2}}{{\mu_{1}}^{2}RR}+\frac{\sigma_{0}^{2}}{{\mu_{0}}^{2}} \right)}} \right)$$

More simply, the power for each of these is:

$$\Phi\left( -1.96-test statistic \right),$$

where a negative test statistic indicates a beneficial effect. $\Phi=Pr(Z\leq z)$ is the cumulative distribution function of a standard normal random variable. Because $\Phi$ is an increasing function, power increases as the magnitude of the (negative) test statistic increases. The formula shows that power increases as:

- n increases
- The control group event rate ($p_{0})$increases
- The negative log HR becomes smaller (more negative, so farther from zero and larger in magnitude), meaning the HR becomes smaller and farther from the null value of 1.
- The negative log ratio of means becomes smaller (more negative, so farther from zero and larger in magnitude), meaning the mean ratio becomes smaller and farther from the null value of 1.
- The risk ratio RR gets closer to zero

Power decreases as:

- the variance of the number of clones at first infection in either arm increases

These formulas obscure the fact that in general, when p_1_ and p_0_ change, the HR also changes. A typical simplification is to assume exponentially distributed event times. Then we have a constant hazard ratio and can calculate the HR as $HR= \frac{\log(1-p_{1})}{\log(1-p_{0})}$. Assuming exponential time to event distributions, we obtain the following power formulas, which rely on p_0_ and p_1_, but not on the HR.

Power for VE_HR_ is:

$$\Phi\left( -1.96-\sqrt{\frac{np_{0}}{2}}\frac{log\frac{\log(1-RRp_{0})}{\log(1-p_{0})}}{\sqrt{\left( 1+\frac{1}{RR} \right)}} \right)$$

As RR gets closer to zero, 1-RRp_0_ gets closer to 1, so log(1-RRp_0_) gets closer to zero, so the log of that gets closer to negative infinity. As p_0_ gets bigger (closer to 1), 1-p_0_ gets closer to zero, so log(1-p_0_) gets closer to negative infinity and so does log(log(1-p_0_)).

Power for VE_c_ is:

$$\Phi\left( -1.96-\sqrt{\frac{np_{0}}{2}}\frac{logHR+log\frac{\mu_{1}}{\mu_{0}}}{\sqrt{\left( 1+\frac{1}{RR}+\frac{\sigma_{1}^{2}}{{\mu_{1}}^{2}RR}+\frac{\sigma_{0}^{2}}{{\mu_{0}}^{2}} \right)}} \right)=\Phi\left( -1.96-\sqrt{\frac{np_{0}}{2}}\frac{log\frac{\log(1-RRp_{0})}{\log(1-p_{0})}+log\frac{\mu_{1}}{\mu_{0}}}{\sqrt{\left( 1+\frac{1}{RR}+\frac{\sigma_{1}^{2}}{{\mu_{1}}^{2}RR}+\frac{\sigma_{0}^{2}}{{\mu_{0}}^{2}} \right)}} \right)$$

The threshold where the difference in power between VE_c_ and VE_HR_ switches from positive to negative is the combination of parameters satisfying:

$$\sqrt{\frac{np_{0}}{2}}\frac{log\frac{\log(1-RRp_{0})}{\log(1-p_{0})}}{\sqrt{\left( 1+\frac{1}{RR} \right)}}= \sqrt{\frac{np_{0}}{2}}\frac{log\frac{\log(1-RRp_{0})}{\log(1-p_{0})}+log\frac{\mu_{1}}{\mu_{0}}}{\sqrt{\left( 1+\frac{1}{RR}+\frac{\sigma_{1}^{2}}{{\mu_{1}}^{2}RR}+\frac{\sigma_{0}^{2}}{{\mu_{0}}^{2}} \right)}}$$

This expression does not reduce to a simple formula. The threshold does not depend on the sample size since the $\sqrt{n}$ on each side drops out of the equation.

The above formulas were validated by estimating power by simulation assuming exponential time-to-event distributions. Rate parameters for time to first infection were chosen to match the infection rates in the control and vaccine groups in the RTS,S data set. Times to first infection were simulated by random draws from the exponential distributions for vaccinees and controls, and those infected after the end of follow-up were censored. The number of clones for infected controls was sampled from its distribution among infected RTS,S control participants, and that for vaccinees was sampled from its distribution among infected RTS,S vaccinees. Power was estimated as the proportion of 1000 simulations for which the null hypothesis was rejected. Power for event rates lower than 0.20 was not estimated as these event rates were generally too low to consistently obtain estimable results and because these ranges are not relevant because power is too low. Figure A5 compares the simulation-based power estimates to those from the formulas and shows very good agreement.

Figure A5. Analytic and simulation-estimated power curves for VE_HR_ and VE_C_ for trials with data structure matching the RTS,S data, assuming exponential time-to-event distributions.


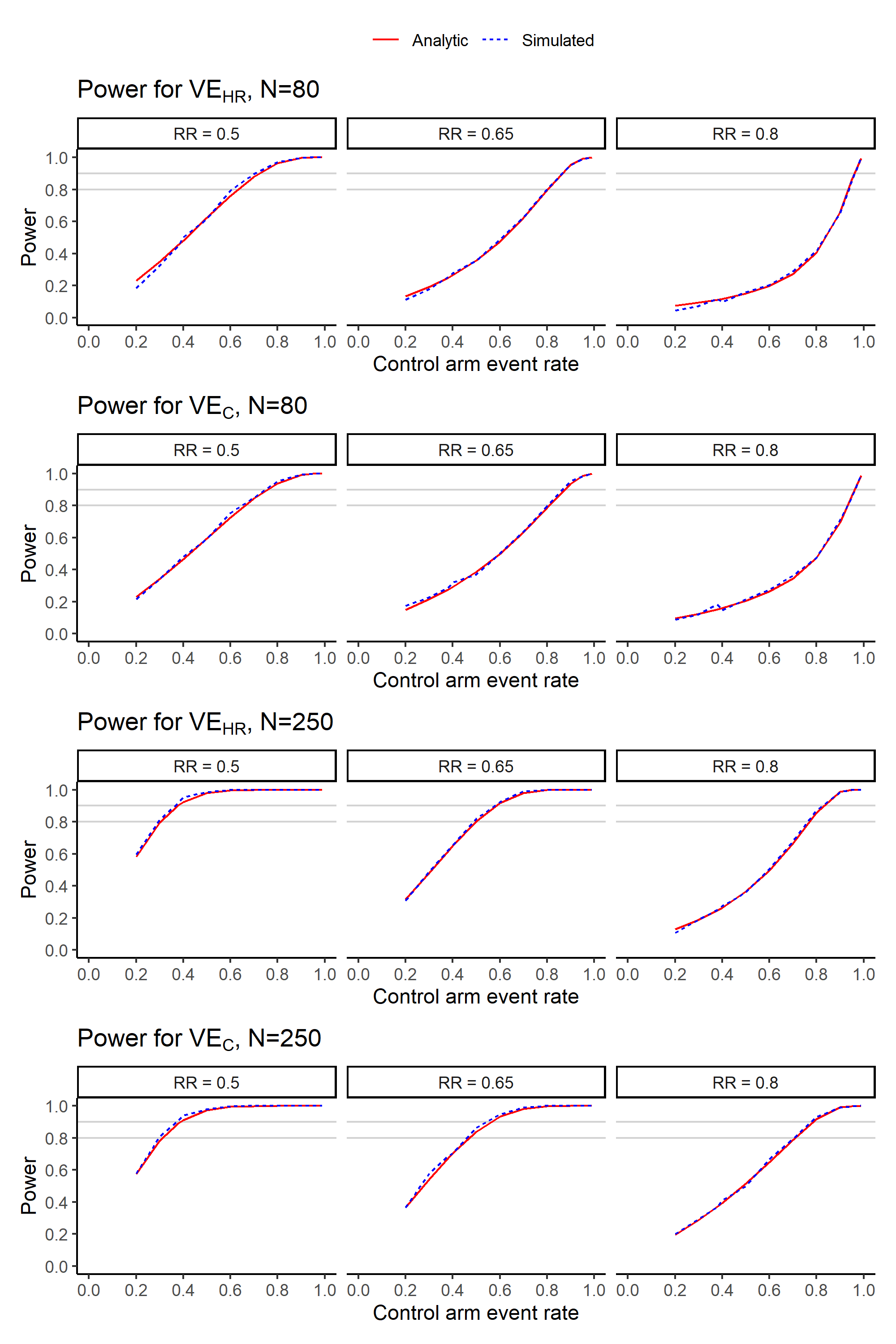


REFERENCES

[1] Jessica Briggs, Noam Teyssier, Joaniter I Nankabirwa, John Rek, Prasanna Jagannathan, Emmanuel Arinaitwe, Teun Bousema, Chris Drakeley, Margaret Murray, Emily Crawford, Nicholas Hathaway, Sarah G Staedke, David Smith, Phillip J Rosenthal, Moses Kamya, Grant Dorsey, Isabel Rodriguez-Barraquer, Bryan Greenhouse (2020) Sex-based differences in clearance of chronic *Plasmodium falciparum* infection eLife 9:e59872

[2] Follmann, Dean, and Chiung‐Yu Huang. "Incorporating founder virus information in vaccine field trials." Biometrics 71.2 (2015): 386-396.

[3] Emil Scosyrev and Ekkehard Glimm. "Power analysis for multivariable Cox regression models." *Statistics in medicine* 38.1 (2019): 88-99.
